# Association of IL-8 and CXCR2 with AST/ALT Ratio in Liver Abnormalities Screening during Oxidative Stress Injury Caused by VCM

**DOI:** 10.1155/2024/1951046

**Published:** 2024-07-30

**Authors:** Yiwen Dong, Xingang Wang, Weijiang Hu, Xin Wang, Hongying Bian, Wencui Zhang, Feng Han, Ning Kang, Lin Zhang, Meng Ye

**Affiliations:** ^1^ Department of Occupational Epidemiology and Risk Assessment Research National Institute for Occupational Health and Poison Control Chinese Center for Disease Control and Prevention, Beijing 100050, China; ^2^ Department of Occupational Health and Radiological Health Center for Disease Control and Prevention in Bin Hai New Area of Tian Jin City, Tian Jin 300480, China; ^3^ Department of Occupational Disease Monitoring and Information Policy Laboratory National Institute for Occupational Health and Poison Control Chinese Center for Disease Control and Prevention, Beijing 100050, China; ^4^ The General Office National Institute for Occupational Health and Poison Control Chinese Center for Disease Control and Prevention, Beijing 100050, China; ^5^ Department of Science and Technology National Institute for Occupational Health and Poison Control Chinese Center for Disease Control and Prevention, Beijing 100050, China

## Abstract

Liver impairment caused by VCM has been linked to irreversible damage such as fibrosis, necrosis, hepatocellular carcinoma, and liver angiosarcoma. However, the ability to detect abnormalities during initial phase have not been achieved so far. Thus, this study aimed to investigate the effect of interleukin 8 (IL-8) and C-X-C chemokines 2 (CXCR2) on screening for a VCM-exposed group (*n* = 227) from a PVC manufacturing factory compared to a control group (*n* = 110) in Tianjin City in 2020 with influence factors evaluation. Ambient concentrations of VCM and health archives from 2012 to 2018 were collected for establishing the dose-effect trend. A cross-sectional survey in 2020 was performed to measure TDGA, IL-8, CXCR2, 8-OHdG, SOD, GPX, CAT, MDA, and ROS levels. Results indicated a continuous increased incidence on liver abnormalities despite a fluctuated downward trend in cumulative time-weighted average (C_TWA_) VCM concentrations over the years. ALT, AST, and AST/ALT ratio all contributed to liver abnormalities that contained fatty liver, liver calcification, and liver cysts, IL-8 and CXCR2 correlated with each other strongly and showed significant associations with oxidative stress markers, even AST/ALT ratio. IL-8 (>1547 *µ*g/m^3^) or CXCR2 (<139 *µ*g/m^3^) influenced the AST/ALT ratio through reciprocal interactions under oxidative stress injury, CXCR2 (>222 *µ*g/m^3^), working years of 21 to 30 (a) and 11 to 20 (a), TDGA (>1.52 mg/L), alcohol consumption, smoking habit, and a less sleeping duration of <4 h per day would also be potential factors affecting the AST/ALT ratio. In conclusion (1) even with decreased VCM concentrations in PVC manufacturing factories liver abnormalities that contained fatty liver, liver calcification, and liver cysts could still occur due to oxidative stress injury with involvement of IL-8 and CXCR2. The status of protective measure and appropriate mask types also play a role; (2) the AST/ALT ratio could be a specific indicator for detecting abnormalities when combined with liver *B* ultrasonography results before impairment altered from bad to worse; and (3) factors such as definite medication history, fully broken protective facilities, alcohol consumption, less sleeping duration, inappropriate mask types, and longer working years could also influence AST/ALT ratio alterations through complex interactions.

## 1. Introduction

Vinyl chloride monomer (VCM) is a volatile organochlorine liquid that typically exists in a gaseous form at room temperature for high volatility. VCM is used primarily for the production of polymer polyvinyl chloride (PVC), with specialties such as water-proofing, anticorrosion, insulation, and plasticity for producing pipes, coating of wire, building materials, and so on [1].

Occupationally, individuals are primarily exposed to ambient VCM through respiratory inhalation. In the liver, VCM is metabolized via the oxidation of CYP2E1, resulting in the formation of a highly reactive intermediate called 2-chloroethylene oxide. This intermediate spontaneously rearranges into 2-chloroacetaldehyde and is further converted to 2-chloroethanol. Finally, the detoxification process involves the action of glutathione and aldehyde dehydrogenase, which convert it into thiodiglycolic acid (TDGA) and N-acetyl-2-(2-hydroxyethyl) cysteine for convenient excretion [2]. It has been proven that high levels of VCM can induce severe health damage. This damage is characterized by acroosteolysis, respiratory irritation on the alveolar epithelium, ataxia, dizziness, and liver impairment. These health effects often involve toxicant-associated steatohepatitis (TASH), peripheral fibrosis, necrosis, hepatocellular carcinoma (HCC), and liver angiosarcoma (ASL) [3].

Technically, acetylene hydrochlorination and ethylene oxychlorination are the two dominant techniques for PVC production. Among them, acetylene hydrochlorination still holds a majority share in the domestic market due to abundant raw materials (calcium carbide ore), low investment requirements, strong technological improvement capability, and independent intellectual property rights that align with the development needs of the coal-electricity-chemical industry.

However, concerns have been raised about the potential health risks associated with VCM exposure, particularly regarding the development of liver abnormalities. Despite efforts to minimize VCM-related health damage, research on the adverse effects of VCM exposure has been ongoing. For example, a case study conducted in 1974 [4] revealed a prevalence of ASL among workers engaged in manual cleaning of PVC polymerization reactors at a plant in Louisville, Kentucky, USA. The study found that ASL occurred after a long latency period (median 36 years) and was predominantly observed in workers with high cumulative VCM exposures (>1000 parts per million (PPM)-years), especially those exposed to ≥10,551 ppm-years who had a hazard ratio of 73.6.

In another study conducted in Porto Marghera, Italy [5], among 1658 workers in a VCM/PVC plant, baggers exposed to VCM were found to have a standardized mortality ratio (SMR) of 1.73 for lung cancer (90% confidence interval 0.93∼3.21), and the SMR ratio between baggers and control posts was 2.31 (90% CI: 1.15∼4.61). These findings highlight the need to investigate whether liver abnormalities can still develop even when current exposure concentrations are below the hygiene limits, as well as the identification of typical imaging results that can facilitate the prompt screening and monitoring of liver abnormalities.

Given that VCM was ranked fourth on the Centers for Disease Control and Prevention's Agency for Toxic Substances and Disease Registry Substance Priority List in 2006 [6], indicating its potential hazards. It has also been classified as a Group I carcinogen by the International Agency for Research on Cancer (IARC) [7, 8], based on animal and occupational epidemiological studies conducted in 2007 and 2012, respectively, which link VCM exposure to HCC and ASL with tangible evidence.

Considering that China's VCM production capacity reached 23.53 million tons in 2018 and is expected to reach 25.93 million tons by 2023 [9], it becomes crucial to prioritize the initial screening for abnormal symptoms or specific indicators, as the health status of workers can often be neglected in high-content and demand environments. One cost-effective method widely used in clinical practice for screening liver abnormalities is liver *B* ultrasonography. This method allows for the identification of conditions such as fatty liver, alcoholic cirrhosis, steatohepatitis, periportal fibrosis with cysts, and even HCC by assessing liver edge bluntness, liver parenchyma coarseness, liver surface nodularity, inferior vena cava irregularity and narrowness, echogenicity, distribution of calcification, and hepatic cysts [10, 11].

Interleukin 8 (IL-8) is a peptide consisting of 72 amino acids that can be secreted by fibroblasts, endothelial cells, epithelial cells, monocytes, macrophages, and cancer cells. It typically signals through two G-protein-coupled receptors, CXCR1 and CXCR2, which belong to the C-X-C chemokine receptor group. Numerous studies have suggested that IL-8 and CXCR1/2 signaling play a role in recruiting neutrophil granulocytes to sites of inflammation; additionally, they contribute to tumor growth by promoting angiogenesis and immune resistance [12].

Moreover, it has been confirmed that the IL-8-CXCR1 signaling pathway primarily promotes cancer cell proliferation in breast cancer, ovarian cancer, and pancreatic cancer [13]. On the other hand, the IL-8-CXCR2 coalition is known to be involved in angiogenesis and the recruitment of neutrophils to the tumor microenvironment [14]. For example, recent studies have shown that the IL-8-CXCR2 axis plays a significant role in the progression of various liver diseases such as alcohol or nonalcohol-related liver disease, hepatitis (HBV/HCV), fibrosis, and cirrhosis [15]. Additionally, liver hepatocytes and resident immune cells can sense changes in the environment and signals in the bloodstream [16]. Once activated by antigens or external insults, CXCR2 and IL-8 are released from the liver to recruit neutrophils, leading to neutrophil-derived oxidative bursts and release of cytotoxic granules, ultimately causing hepatotoxicity and hepatocellular death [17, 18]. In this regard, it is speculated that alterations in the IL-8-CXCR2 interaction may contribute to liver impairment through involvement in oxidative stress injury. Therefore, it is of interest to investigate whether IL-8 and CXCR2 play potential roles or regulate specific indicators during the initial stage of liver impairment under exposure to VCM.

In order to investigate this hypothesis, the objective of this study was to examine the impact of IL-8 and CXCR2 on liver abnormalities and identify potential correlations with health indicators during the initial screening for liver impairments. Furthermore, this study will also analyze other factors that may be crucial in identifying key risk factors in workplace environments. The ultimate goal is to provide effective intervention measures and establish regulated health surveillance for individuals exposed to VCM.

## 2. Materials and Methods

### 2.1. Study Design and Population

A cross-sectional survey that focused on the VCM exposed population was conducted in 2020 in a large-scale PVC manufacturing factory (referred to as G) located in Bin Hai New Area, Tianjin City, China. During the initial stage, a total of 227 workers from 8 major positions in the VCM manufacturing facilities were identified as the exposed group. In comparison, 110 operators from a local canned food processing plant (referred to as H) with no history of chemical hazard exposure were selected as the control group based on specific inclusion and exclusion criteria.

All subjects from either group G or group H had to meet the following requirements: (1) age between 20 and 60 years with no gender limitations; (2) a minimum length of employment in the current factory of at least 1 year and a minimum current working duration of 3 months; (3) involvement in tasks including but not limited to patrol or field operation. Conversely, workers were excluded if they met any of the following criteria: (1) a history of hepatitis virus B or C infection; (2) clinical diagnosis of alcoholic fatty liver, alcoholic liver cirrhosis, or other liver impairments caused from alcohol abuse, trauma, or complicating diseases; (3) a past or present medical history of chronic renal or liver disease, cardiovascular and cerebrovascular diseases such as hypertension, type 2 diabetes, heart disease, and hepatobiliary calculus; (4) incomplete information or lack of health data; and (5) refusal to provide biological specimens or sign informed consent.

The ethical approval for this study was obtained from the Medical Ethics Committee of the National Institute of Occupational Health and Poison Control, Chinese Center for Disease Control and Prevention in Beijing, China (NIOHP202119). Participants who expressed their willingness to take part in the study were informed about the purpose and detailed implementation steps and were required to sign a written informed consent. Furthermore, all methods conducted in this study were in accordance with the relevant guidelines and regulations of the Helsinki Declaration and were approved by the academic board of the National Institute of Occupational Health and Poison Control, Chinese Center for Disease Control and Prevention.

### 2.2. Manufacturing Process Investigation in G and H

**Figure 1:**
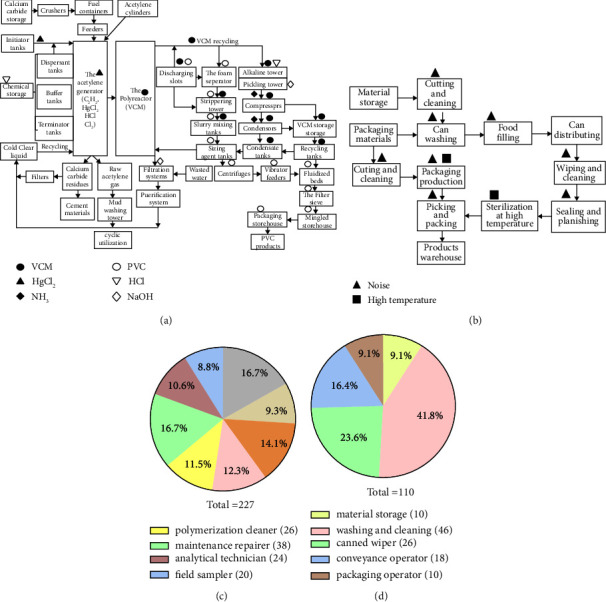
Charts (a and b) display the manufacturing processes and hazardous factors in G and H; charts (c and d) describe the constituent ratios (*R*%) and relevant amounts of different posts. The manufacturing technique of acetylene hydrochlorination could be summarized in following steps. Acetylene (C_2_H_2_) was created by a chemical reaction between water and CaC_2_, VCM was composited through reactions between C_2_H_2_ and HCl under a catalytic action of HgCl_2_, and then PVC was finally produced through VCM polymerization under high temperature and pressure. Partial VCM and waste water were recycled and purified for repeated utilization in (a). Hazardous factors included VCM, PVC (particle state), HCl (36∼38%, pH < 2), NaOH (3.5%, pH = 13.9), HgCl_2_ (solid-state), Cl_2_ (99.9%, liquid state), and NH_3_ (98.5%, liquid ammonia). In (b), the whole process could be illustrated as raw material cleaning and cutting, can filling with automatic machines and exhausting gases from cans or jars, sealing cans with iron caps and sending to sterilization, finally packaging and quality examining, hazardous factors including noise and high temperature. In (c), posts in G were classified into synthetic operators (*n* = 38), refrigerating operators (*n* = 21), aggregated operators (*n* = 32), stripping operators (*n* = 28), polymerization cleaners (*n* = 26), maintenance repairers (*n* = 38), analytical technicians (*n* = 24), and field samplers (*n* = 20). In (d), posts in H involved material storage operators (*n* = 10), washing and cleaning operators (*n* = 46), canned wipers (*n* = 26), conveyance operators (*n* = 18), and packaging operators (*n* = 10).

Factory G is primarily involved in the production of VCM, with an annual production capacity of 800,000 tons, which is further processed into PVC at a rate of 1,000,000 tons per year by using the acetylene hydrochlorination technique. The overall process can be described in the following steps: (1) acetylene is generated through a chemical reaction between water and broken calcium carbide; (2) VCM is synthesized by reacting acetylene with hydrogen chloride under the catalytic influence of mercuric chloride (HgCl_2_); (3) finally, PVC is produced through the VCM polymerization carried out under high temperature and pressure. In contrast, Factory H specializes in the production of canned food, including yellow peaches, pineapples and other fruits. The processes involved in the production can be described as follows: (4) raw materials such as fruits and vegetables are stripped, cleaned and cut; (5) The cans or jars are filled using automatic machines and any gases are exhausted; (6) the cans are sealed with iron caps and subjected to sterilization; and (7) packaging and quality examination are conducted before the products are shipped. Figures 1(a) and 1(b) display the process flow diagrams for Factories G and H, respectively.

### 2.3. Questionnaire Survey

During the physical examination process in 2020, a questionnaire interview was conducted in a face-to-face manner. All participants who voluntarily agreed to take part in the study were provided with information about the purpose and gave their consent. The interviewers were trained in advance to ensure objective inquiry and minimize subjective bias. The questionnaire covered various aspects, including: gender (male/female); age (∼30/∼45/∼60a); working years (∼10/∼20/∼30a); medication history (yes/no)]; smoking habits (yes/no); alcohol consumption (yes/no); sleeping duration (∼4/∼6/∼8 h); weekly exposure time (∼20/∼40 h); mask types [antipoison/antiparticulate/disposable ones; frequency of wearing [always/certain times (especially on feeding raw materials, cleaning up working environment or sampling)]; mask replacement frequency [once per day/infrequently (switching frequency that lower than 3 times per week)]; and protective facility status (normally operating, temporary suspending, and fully broken). These details were collected to ensure a thorough understanding of the participants' backgrounds and work environments.

### 2.4. Health Data Collection

A comprehensive collection of health data and medical records was carried out, encompassing various parameters. These included white blood cell counts (WBC) ranging from 4.0 to 10.0 × 10^9^/L, red blood cell counts (RBC) ranging from 4.5 to 5.5 × 10^12^/L for males and 3.5 to 5.0 × 10^12^/L for females, hemoglobin levels (HGB) ranging from 120 to 160 g/L for males and 110 to 150 g/L for females, blood platelet counts (PLT) ranging from 100 to 300 × 10^9^/L. Furthermore, hepatic function indexes were measured, including alanine aminotransferase (ALT) levels ranging from 5 to 40 U/L, glutamyl transpeptidase (*γ*-GGT) levels ranging from 0 to 50 U/L, aspartic transaminase (AST) levels ranging from 8 to 40 U/L, and AST/ALT ratio should be less than 1.15. Hemobilirubin (TBIL) levels ranged from 3.4 to 17.1 *μ*mol/L, total protein (TP) levels ranged from 60 to 80 g/L, globulin (Glb) levels ranged from 20 to 30 g/L, and albumin (Alb) levels ranged from 35 to 50 g/L. Additionally, blood biochemistry assessments measured total cholesterol (TC) levels, which should be less than 5.2 mmol/L, and triglyceride (TG) levels, which should be less than 1.7 mmol/L. These measurements were conducted biennially from 2012 to 2018. In 2020, other indices such as alkaline phosphatase (ALP) levels ranging from 4 to 150 U/L, absolute neutrophil count (ANC) ranging from 2.0 to 4.0 × 10^9^/L and fasting blood glucose (FBG) ranging from 3.9 to 6.1 mmol/L were included. Moreover, the results from echo-color Doppler ultrasonography tests (referred to as liver *B* ultrasonography test) conducted in 2012, 2014, 2016, and 2018 were further elaborated in 2020. The classifications were expanded to include fatty liver (ranging from moderate to severe degrees), inner-hepatic calcification with thickened echoes (liver calcification), and multiple hepatic cysts (liver cysts). However, only in 2020 were matched information on liver *B* ultrasonography results and health data available. Detection results of C_TWA_ (presumably referring to occupational hazards) among 8 posts were obtained from assessment reports spanning the years 2012 to 2018.

### 2.5. Biological Specimen Sampling and Treatment

In 2020, peripheral venous blood and preshift urine samples were collected from all subjects. Specifically, postshift blood samples were obtained in a fasting state by skilled nurses using anticoagulant-free vacuum collection tubes (BD, USA) at a volume of 4 mL per person. These samples were then centrifuged at 2500 rpm/min for 5 minutes to separate the serum from cellular components. Urine samples, ranging from 10 to 50 mL per individual, were collected through self-collection methods. Immediately after collection, the urine samples were placed in centrifuge tubes at a volume of 15 mL per person and stored at low temperatures until analysis of TDGA content.

### 2.6. VCM Concentration Detection in 2020

Ambient VCM concentration was sampled by using individual air-flow pumps (Casella VAPex/VAPexPRO, UK) at a range of 100–500 mL/min. The pumps were equipped with activated carbon tubes (100 mg each) made from coconut materials (SKC USA). Sampling was conducted among 8 posts with a flow rate of 50 mL/min over a period of 8 hours. The sampling protocols and quantities followed the standards outlined in GBZ 159–2004 (Sampling Practices for Monitoring Harmful Substances in Workplace Air) and GBZ/T 300.1–2017 (Measurement Methods for Toxic Substances in Workplace Air Part 1: general principles), which required the collection of 3 samples per post for three full working shifts or a 12-hour early shift, followed by another 12-hour late shift. After sampling, both ends of the activated carbon tubes and two blank samples were sealed and immediately stored in clean containers until analysis. The treatment steps were as follows: (1) The sampling tubes were placed in a thermal desorption device and heated to 250°C. The air inlet end was connected to a syringe while the other end was connected to a carrier gas (nitrogen) flowing at a rate of 50 mL/min. (2) A series of standard gas samples containing VCM was prepared by diluting national standard material (with a mass of 2.60 mg per 1 mL of VCM gas) in a 100 mL volume syringe to achieve concentrations ranging from 0.0 to 0.30 *µ*g/mL. (3) The gas chromatography instrument (Agilent 6890 N, Agilent Co., Ltd., USA) was optimized in advance, with an injection volume of 1 mL, to detect specific peak heights or peak areas within the standard series. (4) The standard curve and regression equation were generated based on the peak heights or peak areas for calculating the practical concentration. The (1) was used to display the regression equation:

(1) $$\begin{array}{c} C=\frac{C_{0}}{V_{0}\times D}\times 100. \end{array}$$

In the context of this study, the notation used for various parameters is as follows: C represents the ambient concentration of VCM in milligrams per cubic meter (mg/m^3^), *C*_0_ represents the concentration of VCM within the measured samples in micrograms per milliliter (*μ*g/mL), *V*_0_ represents the standardized sampling volume in liters (L), and D represents the desorption efficiency in percentage (%). The entire set of operating steps followed the guidelines outlined in GBZ/T 300.78–2017 (determination of toxic substances in workplace air-Part 78: vinyl chloride, dichloroethene, trichloroethene, and tetrachloroethene) [19].

### 2.7. ELISA Detection

The levels of IL-8, CXCR2, 8-OHdG, GPX, CAT, MDA, SOD, and ROS were measured using ELISA kits for human (Fusheng Biotechnology LTD., Shanghai, China) following the provided protocols. The experimental steps were as follows: The liquid supernate was collected by centrifuging the samples at 2500 rpm/min for 20 minutes after allowing them to stand at room temperature for 20 minutes. A high-speed centrifuge (ST8 Thermo, USA) was used for this step. A 96-well plate was prepared, with blank control wells containing 50 *μ*L of deionized water each, standard substance wells containing a series of concentrations (6.25, 12.5, 25, 50, and 100 ng/L) for 50 *μ*L per well and tested sample wells containing 40 *μ*L of sample diluent and 10 *μ*L of the samples. The plate with all the samples was sealed and incubated at 37°C for 30 minutes in an electrothermal blowing dry box (DHG-9140A, Beijing, China). This step was repeated five times with each repetition involving washing, discarding the liquid, and spinning dry using the scrubbing solution. After thorough washing, 50 *μ*L of the enzyme-labeled reagent was added to each sample well in addition to blank control wells. The plate was sealed again and incubated for another 30 minutes at 37°C. Following the completion of the secondary incubation, the reagent was discarded and the washing steps were repeated five times. Chromogenic reagent A and B were added sequentially to each well, and the plate was kept at 37°C away from light for 10 minutes to allow the chromogenic reaction to occur. Once color development was observed, the reaction was terminated by adding 50 *μ*L of stop buffer to each well. The absorbance of the wells was measured at a wavelength of 450 nm using a microplate reader (Multiskan™FC, Thermal, USA). The content of each index was then calculated using a linear regression equation derived from the relevant standard series.

### 2.8. Urinal TDGA Detection

TDGA content was determined using solid phase extraction ion chromatography, following the specified procedures [20]. The operational steps were as follows: Sequentially, 2 mL of chromatographically pure methanol (Fisher Scientific, USA) and 2 mL of deionized water (>1822 MΩ·cm) were added to C18 solid phase extraction columns (200 g and 3 mL per column, Agilent, USA). The columns were then further activated through natural leaching with cleaned residential liquid. Urine specimens (15 mL volume per column) were centrifuged at 3000 r/min for 10 minutes. From the resulting supernatant, 2.5 mL was extracted and titrated into a 15 mL centrifuge tube (CORNING, USA) with deionized water to scale. After shaking and mixing for 10 minutes, 2.0 mL of the diluted urine was passed through activated C18 columns. The filtering liquid was fully collected and passed through a needle-type filtering head (pore size 0.22 *μ*m, Zenten, Germany) once more. The collected filtering liquid was used for loading. Detailed steps for chromatographic column analysis (LonPac ASl9, 250 mm × 4 mm) were followed, the mobile phase for column protection to IonPae AGl9 was KOH liquid, which was created by an in-line generator with leaching liquid. The gradient leaching pattern (with a flow rate of 1.0 mL/min) consisted of 20 mmol/L for 0–10 minutes, 20–30 mmol/L for 10–20 minutes, 30–60 mmol/L for 20–30 minutes, and 60 mmol/L for 30–36 minutes. The current for the suppressor was set at 150 mA and the automatic injection quantity was limited to 25 *μ*L. Standard series were prepared using the following steps: Precisely weighing 50 mg of TDGA standard material (Dr. Ehrenstorfer GmbH, Germany) and making it into a standardized stock solution of 1000 *μ*g/mL. Diluting the stock solution further to prepare a standard series of 100 *μ*g/mL (with concentrations of 0, 0.05, 0.10, 0.50, 2.50, 10.00, and 50.00 *μ*g/mL). The injection quantity was limited to 25 *μ*L, and results were determined by retention time (21.13 min) and quantified based on peak area. Equation (2) is represented as follows:

(2) $$\begin{array}{c} C=C_{0}\times 6, \end{array}$$

where *C* represents the mass concentration (*μ*g/mL) of TDGA in urine. C_0_ represents the mass concentration (*μ*g/mL) of TDGA detected through solid phase extraction ion chromatography. The coefficient of 6 represents the dilution factor of 6 times.

### 2.9. Statistical Analysis

*Epidata 3.0* and *SPSS 24.0* (SPSS Inc., Chicago, IL, USA) were utilized for database establishment and statistical analysis. Data normality was tested with the one-sample *Kolmogorov‒Smirnov* method. For the normally distributed data, the *Student's t test* was used; otherwise, the *Mann‒Whitney U* nonparametric test was used instead. The *X*^2^ test was utilized to compare differences in the composition rate (%) of liver *B* ultrasonography results in questionnaire analysis. *Spearman* correlation analysis was done for variable correlations among abnormal distribution data. The contribution of independent variants to certain dependent variants was carried out through the linear regression model and a generalized linear model was implemented to determine potential interactions among variables while potential confounding factors were controlled. Multivariate ANOVA was utilized to explore possible independent variables that contributed to multiple dependent variables, the interactive effect between bilateral variables was determined through *LSD*. Statistical significance for two-tailed *P* values was defined as *α* < 0.05 or <0.001.

## 3. Results

### 3.1. Occupational Field Investigation

Critical risk points in facility G include material warehouses, acetylene gas generators, VCM reaction towers, condensing apparatus, high and low boiling towers, polymeric kettles as well as gas stripping towers. The identified hazardous factors in these areas containing VCM, PVC (in particulate state), HCl (36–38%, pH < 2), NaOH (3.5%, pH = 13.9), HgCl_2_ (solid-state), Cl_2_ (99.9%, liquid state), and NH_3_ (98.5%, liquid ammonia). The typical job positions involved in these processes including synthetic operators, refrigerating operators, aggregated operators, stripping operators, polymerization cleaners, maintenance repairers, analytical technicians, and field samplers. Their tasks usually involve in instrument operation, on-site patrolling, safety inspection, product sampling, and reaction observation. The working shifts in this facility consist of 8-hour shifts (day shift: 8:00am–3:30pm, midnight shift: 4:00pm–11:30pm, and night shift: 12:00am–8:00am) as well as 12-hour shifts (early shift: 8:30am–7:30pm and late shift: 8:00pm–8:00am). During their work, all employees in G are required to wear facial masks or respirators to protect against dust and chemical hazards. They also wear earplugs and helmets as necessary. In contrast, facility H relies heavily on a semiautomatic production line combined with manual operations. The processes in H including iron sheet cutting, cleaning and disinfection, filling food, sealing, and packaging. The workers in H are responsible for tasks such as material storage, washing and cleaning, can wiping, conveyance, and packaging. These processes are associated with environmental noise, high temperature, and local vibration. Similar to G, the workers in H also follow the same shift patterns and use individual protective tools. The process flow charts and pie charts illustrating the distribution of job positions in G and H are presented in Figures 1(a), 1(b), 1(c), and 1(d).

### 3.2. Demographic Characteristics and Questionnaire Analysis

**Figure 2:**
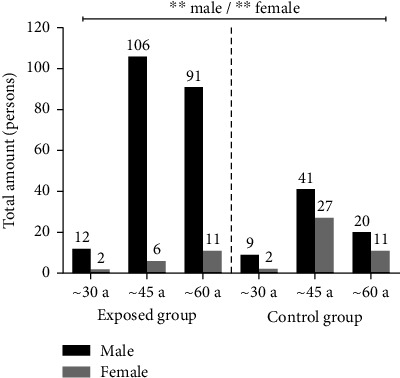
This chart presented significant differences in age distribution (∼30a,∼45a,∼60a) of gender between the two groups. In that, annotation indicated the quantity of people among each age range in either sex, ^*∗∗*^indicates *P* < 0.05.

As shown in Table 1, significant differences in average ages (mean ± SD) were only found between male workers in two groups (*t* = 4.52, *P* < 0.001). Noteworthy differences were also observed in working years, with males (*t* = 6.98, *P* < 0.001) and females (*t* = 8.69, *P* < 0.001) showing differences between groups.  Figure 2 indicates that significant differences were only seen in the population distribution within each sex between groups (*X*^2^ = 7.10, *P*=0.029 for males; *X*^2^ = 6.74, *P*=0.034 for females). Thus, it could be concluded that the two groups have similar age and gender distributions, making them comparable. Next, statistical differences among liver abnormalities, including normal, fatty liver, liver calcification, and liver cysts, were analyzed using the chi-square test after adjusting for demographic characteristics, lifestyle habits, occupational history, and protective measures related to masks and facilities. Table 1 also presented significant differences in the population quantity for variables such as smoking habits (*X*^2^ = 12.97, ^*∗∗*^*P* < 0.05), working years (*X*^2^ = 15.85, ^*∗∗*^*P* < 0.05), weekly exposure time (*X*^2^ = 15.85, ^*∗∗*^*P* < 0.05), mask types (*X*^2^ = 44.78, ^*∗∗∗∗*^*P* < 0.001), frequency of wearing (*X*^2^ = 15.34, ^*∗∗*^*P* < 0.05), mask replacement frequency (*X*^2^ = 8.82, ^*∗∗*^*P* < 0.05), and protective facilities status (*X*^2^ = 65.07, ^*∗∗∗∗*^*P* < 0.001) in the exposed group (^*∗∗*^*P* < 0.05, ^*∗∗∗∗*^*P* < 0.001). Similarly, the control group shows notable differences in variables such as medication history (*X*^2^ = 18.74, ^*∗∗∗∗*^*P* < 0.001) and sleeping duration (*X*^2^ = 20.34, ^*∗∗*^*P* < 0.05). Further analysis revealed significant differences between the two groups in terms of the quantity of males, definite medication history (yes), smoking habits (yes), working years in the range of 10–20 years, age range of 20–30 years, wearing disposable masks and having a normal protective facility status (^*∗∗*^*P* < 0.05). Therefore, it could be assumed that besides exposure to VCM, factors such as definite medication history, sleeping duration (4–6 hours per day/≤4 hours per day), male gender, smoking habits, age range of 20–30 years, working years of 11–20 years and 21–30 years, longer weekly exposure time (20−40 hours), inappropriate mask wearing, nonstandardized wearing actions, irregular mask replacement frequencies, and inadequate maintenance of protective facilities might also contribute to liver abnormalities.

Results of the multiple logistic regression analysis of liver *B* ultrasonography in 2020 between groups are presented in Table 2. It was revealed that alcohol consumption was mainly associated with fatty liver (Exp(*B*) = 1.288, 95% CI = 1.071∼2.179), liver calcification (Exp(*B*) = 1.592, 95% CI = 1.277∼3.063), and liver cysts (Exp(*B*) = 1.801, 95% CI = 1.030∼3.483) in the exposed group, as well as fatty liver (Exp(*B*) = 2.421, 95% CI = 1.141∼4.003), and liver calcification (Exp(*B*) = 1.761, 95% CI = 1.161∼3.323) in the control group. Working years were associated with fatty liver (∼20a, Exp(*B*) = 1.488, 95% CI = 1.161∼2.310; ∼30a, Exp(*B*) = 1.626, 95% CI = 1.252∼2.861) and liver calcification (∼20a, Exp(*B*) = 1.116, 95% CI = 1.020∼1.420; ∼30a, Exp(*B*) = 1.406, 95% CI = 1.043∼1.895) in the exposed group, but not in the control group. Furthermore, infrequent mask replacement (Exp(*B*) = 1.004, 95% CI = 0.932∼1.321), using disposable masks (Exp(*B*) = 1.026, 95% CI = 0.901∼1.906) and recent sleeping duration of 4–6 hours per day (Exp(*B*) = 2.059, 95% CI = 1.129∼4.615) or less than 4 hours per day (Exp(*B*) = 1.249, 95% CI = 1.154∼3.801) were associated with liver calcification in the exposed group. On the other hand, being male and having a medical history were associated with fatty liver (Exp(*B*) = 1.047, 95% CI = 0.971∼1.129) and liver calcification (Exp(*B*) = 1.329, 95% CI = 1.067∼1.620) in the control group with statistically significance (^*∗∗*^*P* < 0.05). Thus, based on the results, alcohol consumption, working years of 10–20 years and 20–30 years, infrequent mask replacement, using disposable masks, sleeping duration of 4–6 hours per day or less than 4 hours per day, being male as well as definite medical history might be all factors to impact liver abnormalities.

### 3.3. Retrospective Analysis on Health Data and VCM Concentrations from 2012 to 2020

**Figure 3:**
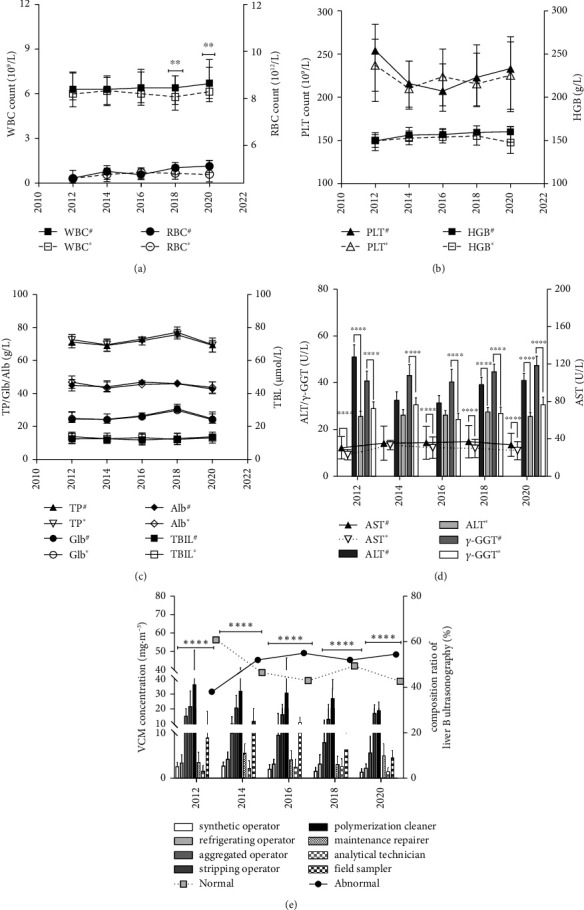
Curve charts (a–d) presented the tendencies and potential differences between workers in G who were classified as “normal (^*∗*^)” and those classified as “abnormal (^#^)” among indicators such as WBC, RBC HGB, PLT, TP, Glb, Alb, TBIL, ALT, AST, *γ*-GGT, and AST/ALT ratio in 2012, 2014, 2016, 2018, and 2020, respectively, ^*∗∗∗∗*^indicated *P* < 0.001, ^*∗∗*^indicated *P* < 0.05. Diagram (e) illustrated tendencies of VCM concentrations (CTWA) among 8 posts in the exposed group and composition rate (*r*%) for liver ultrasonography results of “normal” and “abnormal” at 2012, 2014, 2016, 2018, and 2020 as^*∗∗∗∗*^indicates *P* < 0.001.

Individual health data and VCM concentration (C_TWA_) among different years (2012, 2014, 2016, 2018, and 2020) were presented in Figures 3(a), 3(b), 3(c), 3(d), and 3(e). Based on charts A to D, significant differences were observed in indicators of WBC, ALT, AST, *γ*-GGT, and AST/ALT ratio between workers with “normal” and “abnormal” screening results. Content of these indicators was relatively higher in “abnormal” workers compared to “normal” workers. Additionally, from 2012 to 2020, content of ALT, AST, AST/ALT ratio, and *γ*-GGT gradually increased in workers with liver abnormalities compared to “normal” workers. In Figure 3(e), significant differences in VCM concentration among different posts were evident (*F* = 33.94, *P* < 0.001 in 2012; *F* = 21.66, *P* < 0.001 in 2014; *F* = 13.11, *P* < 0.001 in 2016; *F* = 29.19, *P* < 0.001 in 2018; *F* = 26.55, *P* < 0.001 in 2020) over consecutive years. Based on these findings, posts were classified into the high exposed group, including polymerization cleaners [27.4 (18.1, 35.5)] mg/m^3^, stripping operators [15.7 (11.9, 24.2)] mg/m^3^ and aggregated operators [9.1 (4.5, 13.1)] mg/m^3^. The medium-exposed group includes field samplers [6.1 (3.7, 12.4)] mg/m^3^ and maintenance repairers [3.7 (2.3, 6.5)] mg/m^3^. The low-exposed group consisted of refrigerating operators [3.3 (2.0, 4.2)] mg/m^3^, synthetic operators [1.9 (1.0, 2.9)] mg/m^3^ and analytical technicians [1.4 (0.9, 2.8)] mg/m^3^.

Furthermore, a decreasing trend in VCM concentrations among posts of polymerization cleaners, synthetic operators and aggregated operators (from 2012 to 2020), field samplers and refrigerating operators (from 2014 to 2020), stripping operators (from 2012 to 2018), and maintenance repairers (from 2014 to 2018) were observed without statistical significance (*P* > 0.05). However, the composition rate (*r*%) for liver abnormalities increased consistently over the years, reaching 54.4% by 2020 from approximately 38.0% since 2012, with a peak value of 55.0% in 2016. In contrast, the *r*% for “normal” individuals started to decline and fluctuated from 60.8% in 2012 to 42.6% by 2020. These findings indicated that ALT, AST, AST/ALT ratio, and *γ*-GGT in the exposed group were associated with liver abnormalities. The gradually increasing *r*% for abnormalities, coupled with the decreasing trend of VCM concentration among different posts, suggested that liver abnormalities caused by VCM exposure may persist even if the VCM ambient concentration had significantly decreased over the past consecutive years.

### 3.4. Binary Logistic Regression toward Health Data from 2012 to 2018

**Figure 4:**
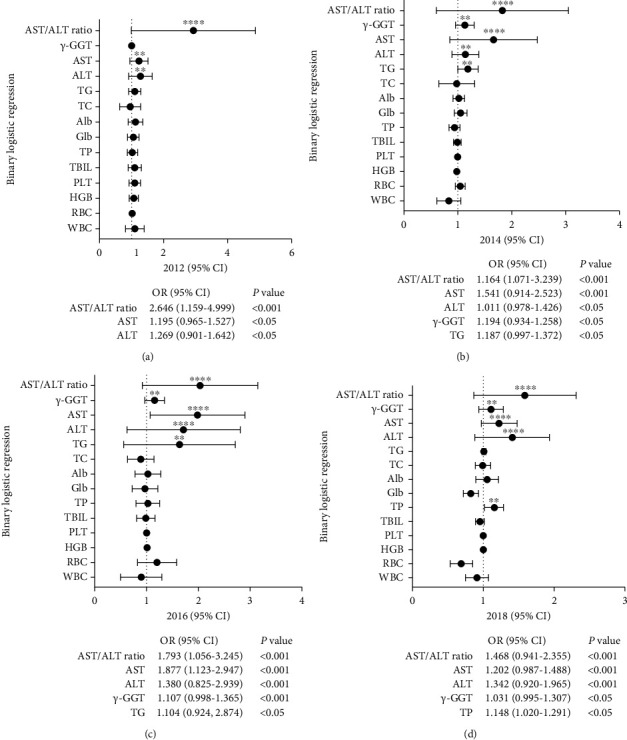
Charts (a–d) demonstrated the 95% CI (upper and lower limits) and OR values for liver *B* ultrasonography results from the exposed group that could only be diagnosed as normal vs abnormal at 2012 (a), 2014 (b), 2016 (c), and 2018 (d) among health indicators by using a binary logistical regression model (^*∗∗∗∗*^*P* < 0.001, ^*∗∗*^*P* < 0.05).

Binary logistic regression was employed to analyze the potential impact of health indicators on liver *B* ultrasonography results (normal vs abnormal) in the exposed group during the years 2012, 2014, 2016, and 2018. Figures 4(a), 4(b), 4(c), and 4(d) illustrated that indicators such as AST/ALT ratio, AST, ALT (from 2012 to 2018), *γ*-GGT (from 2014 to 2018), TG (in 2014 and 2016), and TP (only in 2018) significantly contributed to liver abnormalities (^*∗∗∗∗*^*P* < 0.001 or ^*∗∗*^*P* < 0.05). These indicators usually reflected liver functional status and were served as early signs of hepatocyte injuries, as supported by a review on aminotransferase activity and liver function [21]. Conversely, indicators including TC, Glb, Alb, TBIL, PLT, HGB, RBC, and WBC did not demonstrate any significant contributions to liver abnormalities.

### 3.5. Health Data Analysis between Groups in 2020

The liver *B* ultrasonography results for both groups were further categorized into specific groups, including “normal,” “fatty liver,” “liver calcification,” and “liver cysts” in 2020. The corresponding health data was also stratified accordingly and analyzed using *ANOVA*. In the exposed group, significant differences were observed in the levels of TP (*F* = 5.195, *P* < 0.05), Glb (*F* = 2.758, *P* < 0.05), WBC (*F* = 11.41, *P* < 0.05), RBC (*F* = 21.01, *P* < 0.001), HGB (*F* = 16.61, *P* < 0.001), ANC (*F* = 6.988, *P* < 0.001), FBG (*F* = 11.28, *P* < 0.001), TG (*F* = 35.67, *P* < 0.001), TC (*F* = 23.66, *P* < 0.001), ALT (*F* = 36.47, *P* < 0.001), AST (*F* = 4.676, *P* < 0.05), ALP (*F* = 5.823, *P* < 0.05), *γ*-GGT (*F* = 9.769, *P* < 0.05), and AST/ALT ratio (*F* = 3.569, *P* < 0.05) among the categories of “fatty liver,” “liver calcification,” and “liver cysts” as compared to the “normal” category.

**Figure 5:**
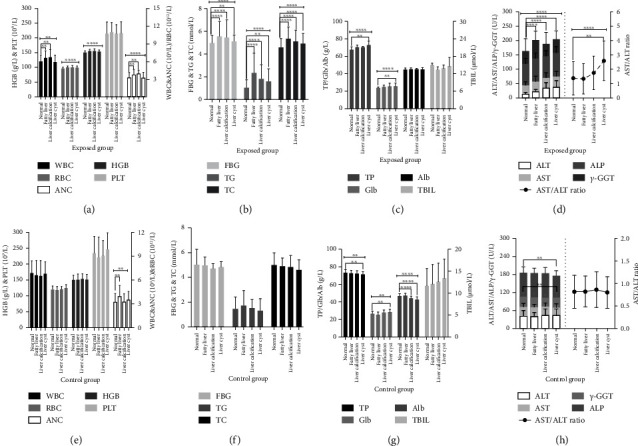
Curve charts (a–d) presented content differences toward WBC, RBC, HGB, PLT, ANC, FBG, TG, TC, TP, Glb, Alb, ALT, AST, ALP, *γ*-GGT, and AST/ALT ratio among categories of “fatty liver,” “liver calcification,” and “liver cysts” compared to “normal” in the exposed group at 2020; curve charts (e–h) equally displayed expression levels of indicators in the control group at 2020, as ^*∗∗∗∗*^indicated *P* < 0.001, ^*∗∗*^meant *P* < 0.05.

Notably, significant differences in WBC and ANC were found between categories of “fatty liver” (*t* = 4.446, *P* < 0.05; *t* = 3.160, *P* < 0.05) and “liver calcification” (*t* = 4.503, *P* < 0.05; *t* = 3.498, *P* < 0.05) as compared to the “normal” category. The levels of FBG (*t* = 5.626, *P* < 0.001; *t* = 4.147, *P* < 0.001; *t* = 2.347, *P* < 0.05), TG (*t* = 9.759, *P* < 0.001; *t* = 7.653, *P* < 0.001; *t* = 5.718, *P* < 0.001) and TC (*t* = 8.356, *P* < 0.001; *t* = 5.812, *P* < 0.001; *t* = 3.668, *P* < 0.05) were highest in the categories of “fatty liver,” “liver calcification,” and “liver cysts” in order (Figure 5(b)). Moreover, there were significant differences in ALT, AST, ALP, *γ*-GGT, and AST/ALT ratio observed across all categories compared to the “normal” category (*P* < 0.001 or *P* < 0.05), as indicated in Figures 5(a), 5(b), 5(c), and 5(d).

In the control group, the *ANOVA* test revealed significant differences in the levels of ANC (*F* = 4.064, *P* < 0.05), TP (*F* = 3.428, *P* < 0.05), and Glb (*F* = 11.01, *P* < 0.05) among all categories, while no such difference was noted in levels of WBC, RBC, HGB, PLT, FBG, TG, TC, ALT, AST, and AST/ALT ratio (Figures 5(e), 5(f), 5(g), and 5(h)). Specifically, slight differences were found in the levels of TP (*t* = 4.320, *P* < 0.05), Glb (*t* = 4.706, *P* < 0.05) and AST (*t* = 2.740, *P* < 0.05) between categories of “liver cysts” and “normal” in Figures 5(g) and 5(h), while similar differences were also observed in ANC (*t* = 4.064, *P* < 0.05), particularly between “fatty liver” and “normal” (Figure 5(e)). Therefore, it could be concluded that indicators such as WBC, RBC, HGB, FBG, TG, TC, ALT, AST, ALP, *γ*-GGT, and AST/ALT ratio exhibited significant differences among specific categories of liver abnormalities in the exposed group. However, ANC, TP, and Glb may not be specific indicators for liver abnormalities, as their differences between “liver cysts” and “normal” were found in both groups.

### 3.6. Multiple Logistic Regression of Health Data between Groups in 2020

**Figure 6:**
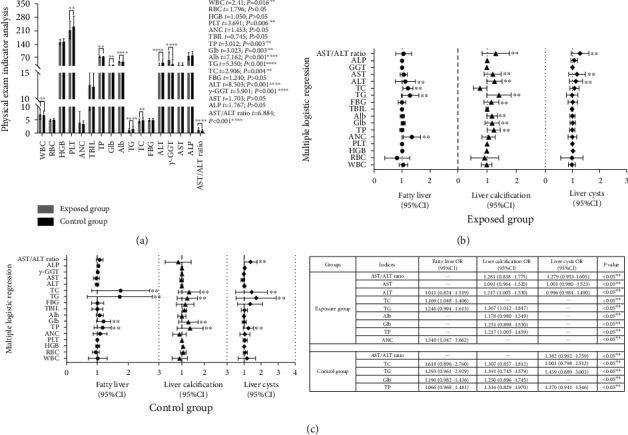
(a–c). Chart (a) demonstrated content differences in health indices, such as WBC, RBC, HGB, PLT, ANC, TBIL, TP, Glb, Alb, TG, TC, FBG, ALT, *γ*-GGT, AST, ALP, and AST/ALT ratio between groups. Chart (b) presented the 95% CI (upper and lower limits) and OR values for live *B* ultrasonography results (categories of “fatty liver,” “liver calcification,” and “liver cysts”) in exposed group in 2020, ^*∗∗*^*P* < 0.05. Chart (c) presented the 95% CI and OR values for relative live *B* ultrasonography results in control group in 2020, ^*∗∗*^*P* < 0.05.

Subsequently, a comparison was made between health data of the two groups in 2020 to identify any differences that could potentially contribute to specific diagnostic categories. In Figure 6(a), significant differences were observed in WBC, PLT, TP, Glb, Alb, TG, TC, ALT, *γ*-GGT, and AST/ALT ratios between the groups. Specifically, in the exposed group, ALT, AST, and AST/ALT ratio played noteworthy roles in “liver calcification” and “liver cysts,” while ALT specifically contributed to “fatty liver” (^*∗∗*^*P* < 0.05). Furthermore, TG, TC, ANC, TP, Glb, and Alb were found to play roles either in “fatty liver” or in “liver calcification” (^*∗∗*^*P* < 0.05), as shown in Figures 6(b) and 6(c). On the other hand, in the control group, TG and TC played significant roles across all categories, while TP and Glb had minimal contributions to “fatty liver” and “liver calcification” (^*∗∗*^*P* < 0.05). Additionally, the AST/ALT ratio and TP showed other contributions to “liver cysts” (^*∗∗*^*P* < 0.05) instead. Thus, it appeared that ALT, AST, and AST/ALT ratio demonstrated significant contributions to abnormality categories under VCM exposure, while TG, TC, ANC, TP, Glb, and Alb may not be specific indicators. Some studies showed that they could be indicators for nonalcoholic fatty liver disease (NAFLD) caused by a high-fat diet (HFD). For example, Lang et al. [22] study on mice exposed to VCM and supplied with an HFD demonstrated that VCM exposure at levels considered safe (OSHA <1 ppm) could worsen experimental NAFLD and led to liver impairment due to secondary insults, such as decreased mitochondrial function and ER stress. These effects can affect mechanisms related to VCM-enhanced HFD-induced liver injury. The significant decrease in mitochondrial activity and subsequent depletion of adenosine triphosphate likely contributed to increased liver injury, elevated production of reactive oxygen species, ultimately led to an increased hepatocyte necrosis.

### 3.7. Correlations between VCM and TDGA Content in 2020

**Figure 7:**
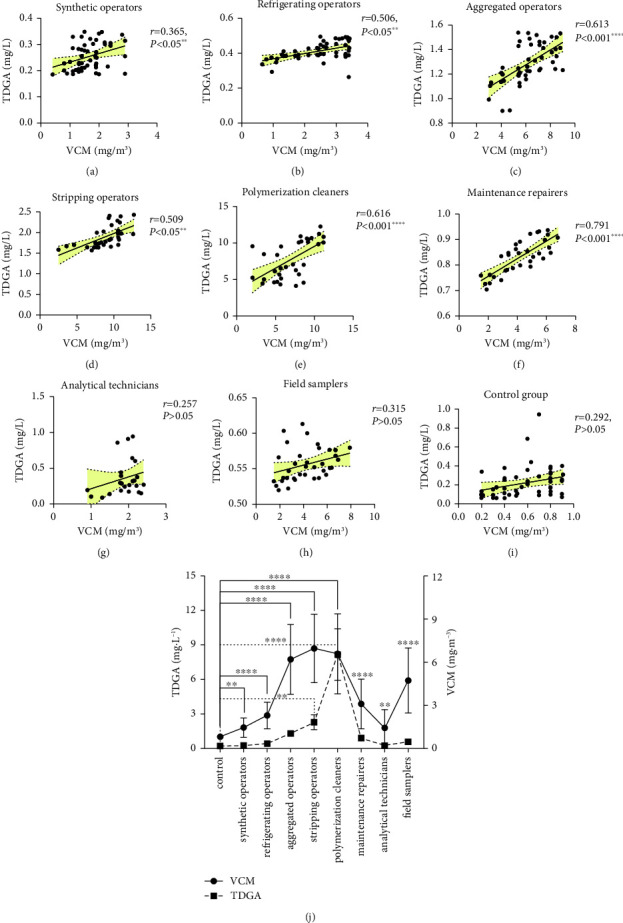
Scatter diagrams (a–i) displayed the correlations between VCM concentration and TDGA content among posts of synthetic operators (a), refrigerating operators (b), aggregated operators (c), stripping operators (d), polymerization cleaners (e), maintenance repairers (f), analytical technicians (g), and field samplers (h), as well as operators in control group (i), and the *r* value indicated correlation intensity coefficient. In that, the strong correlations referred to r > 0.7; the moderated correlation referred to 0.4 < *r* < 0.7; the mild correlation referred to *r* < 0.4 as ^*∗∗*^*P* < 0.05 and ^*∗∗∗∗*^*P* < 0.001. Chart (j) presented significant differences between VCM concentration and TDGA content among posts in exposed group and oriented tendencies among them as ^*∗∗*^*P* < 0.05 and ^*∗∗∗∗*^*P* < 0.001.

A total of 72 VCM samples and 227 urine samples were analyzed in the exposed group, while 15 air samples and 110 urine samples were analyzed in the control group by using relevant methods. Generally, C_TWA_ levels among stripping operators [9.30 (7.70, 10.61)] mg/m^3^ and polymerization cleaners [9.54 (6.24, 11.69)] mg/m^3^ exceeded the OEL level (PC-TWA = 10 mg/m^3^), while results for others fell within the range of 10% to 50% of the OEL or 50% to 1-fold OEL. The TDGA content among posts ranged from an average of 0.65 to 17.84 mg/L. Furthermore, moderate correlations (*r* = 0.365 to 0.616, *P* < 0.05 or <0.001) were observed between VCM and TDGA content in posts of synthetic operators, refrigerating operators, aggregated operators, stripping operators, and polymerization cleaners, whereas a strong correlation was only found in postmaintenance repairers (*r* = 0.719, *P* < 0.001). The fluctuating tendencies of VCM and TDGA were similar, as shown in Figures 7(a), 7(b), 7(c), 7(d), 7(e), 7(f), 7(g), 7(h), 7(i), and 7(j).

### 3.8. Odds Ratio of Liver *B* Ultrasonography Results in 2020

**Figure 8:**
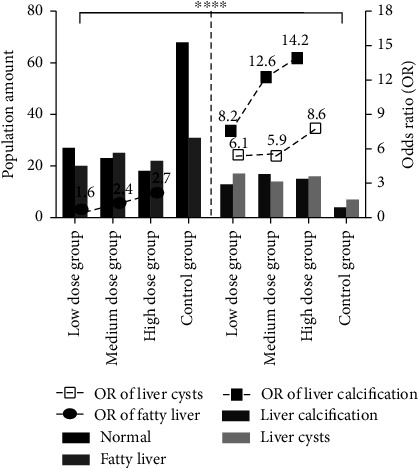
In equation of OR = (^a^/^c^)/(^b^/^d^), ^a^represented those who had abnormal symptoms in exposed group; ^c^represented those who had abnormal symptoms in the control group; ^b^represented those who had no abnormal symptoms in exposed group; ^d^represented those who had no abnormal symptoms in the control group. A significant difference in population size among groups under different categories were analyzed through the chi-square analysis (*X*^2^), as^*∗∗∗∗*^represented *P* < 0.001.

The risk of specific liver abnormalities in different exposure groups was assessed by using odds ratios (OR). To calculate the OR, the numbers of workers with liver abnormalities (a) and without liver abnormalities (c) were compared to ones with liver abnormalities (b) and without liver abnormalities (d) in the “normal” group. The OR values for workers in the low-, medium-, and high-exposure groups were found to be 1.6-fold, 2.4-fold, and 2.6-fold higher for “fatty liver,” respectively, 8.2-fold, 12.6-fold, and 14.2-fold higher for “liver calcification,” and 6.1-fold, 5.9-fold, and 8.6-fold higher for “liver cysts,” respectively, as compared to those in the “normal” group. The most significant increase was observed in the incidence of “liver calcification,” followed by “fatty liver” and “liver cysts” in order. In addition, a significant difference in population size was observed among the different exposure groups by using the chi-square analysis (*X*^2^ = 44.32, *P* < 0.001), as shown in Figure 8. All these suggested that risks of developing liver calcification, liver cysts, and fatty liver in the exposed group were closely associated with VCM exposure levels as the increased concentration of VCM may serve as a contributing factor to the rising incidence of liver abnormalities.

### 3.9. Detection of IL-8, CXCR2, and Oxidative Stress Indices

**Figure 9:**
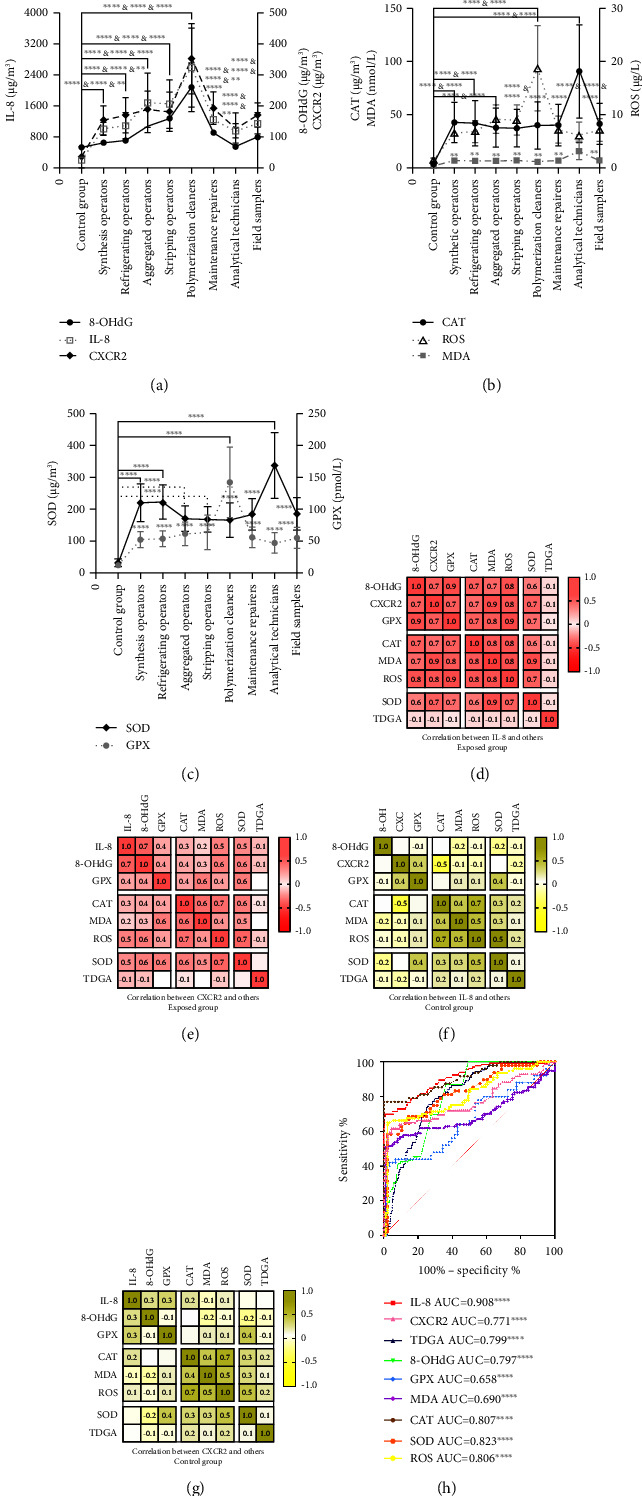
Charts (a–c) displayed differences in content of IL-8, CXCR2, 8-OHdG, SOD, GPX, CAT, MDA, and ROS among 8 posts in exposed group in comparison to workers in control group, as ^*∗∗*^*P* < 0.05 and ^*∗∗∗∗*^*P* < 0.001. Chart (h) presented the ROC curve for judgement towards applicable indices between groups. The value of AUC would be regarded as valuable ones when it approached to 1.0 (0.5∼1.0). Charts (d and e) demonstrated the correlation matrix among IL-8, CXCR2, and TDGA with oxidative stress indices in exposed group. Charts (f and g) similarly demonstrated the correlation matrix among IL-8, CXCR2, and TDGA with oxidative stress indices in control group, *r* referred to the correlation intensity coefficient (*r* < 0.4 for mild intensity, 0.4 < *r* < 0.7 for moderate intensity, *r* > 0.7 for strong intensity).

Figures 9(a), 9(b), and 9(c) illustrate significant differences in the levels of IL-8, CXCR2, 8-OHdG, SOD, GPX, CAT, MDA, and ROS between posts and the control group (^*∗∗*^*P* < 0.05, ^*∗∗∗∗*^*P* < 0.001). Specifically, posts occupied by aggregated operators, stripping operators and polymerization cleaners showed the highest levels of IL-8 (1223.9∼3223.4 *µ*g/m^3^), CXCR2 (152.1∼486.6 *µ*g/m^3^), 8-OHdG (126.8∼328.8 *µ*g/m^3^), ROS (7.26∼24.6 *µ*g/L), and GPX (47.1∼185.6 pmol/L) then followed by posts from medium- and low-exposure groups. However, a contrasting trend was observed for SOD, CAT, and MDA levels in posts occupied by analytical technicians [SOD (226.8∼533.8) *µ*g/m^3^, CAT (53.8∼133.6) *µ*g/m^3^, MDA (7.82∼20.41) nmol/L], synthetic operators [SOD (183.1∼245.9) *µ*g/m^3^, CAT (31.3∼46.9) *µ*g/m^3^, MDA (6.13∼8.26) nmol/L], and refrigerating operators [SOD (174.2∼261.4) *µ*g/m^3^, CAT (28.6∼46.0) *µ*g/m^3^, MDA (5.79∼8.32) nmol/L]. In contrast, posts held by aggregated operators, stripping operators, and polymerization cleaners in the high-exposure group showed lower levels, probably indicated significant enzyme consumption during antioxidative processes under VCM exposure.

Guardiola et al. [23] investigated a combined effect of diet-induced liver injury and VCM exposure in mice. Their study found that an imbalance in the hepatic oxidative defense system induced by toxicants could lead to hepatic inflammation. Liver defense enzymes showed that VCM exposure significantly depleted hepatic superoxide dismutase 3 (SOD3) and heme oxygenase 1 (HO1), but had insignificant effects on SOD1, SOD2, CAT, nuclear factor erythroid-derived 2 (NRF2), and MDA levels. These suggested that levels of IL-8 and CXCR2 in the exposed group were elevated in response to increased VCM concentrations, which indicating the extent of oxidative stress injury and changes in antioxidative enzymes.

Furthermore, a ROC curve in Figure 9(h) demonstrates that IL-8 had the highest area under the curve (AUC) value of 0.908 (*P* < 0.001), followed by SOD (0.823, *P* < 0.001), CAT (0.807, *P* < 0.001), ROS (0.806, *P* < 0.001), TDGA (0.799, *P* < 0.001), 8-OHdG (0.797, *P* < 0.001), and CXCR2 (0.771, *P* < 0.001). GPX (0.658, *P* < 0.05), and MDA (0.690, *P* < 0.05) had relatively lower AUC values. Thus, it could be inferred that IL-8, SOD, CAT, ROS, TDGA, 8-OHdG, and CXCR2 were potential indicators that significantly differentiated between the two groups.

### 3.10. Correlations of IL-8 and CXCR2 with Oxidative Stress Indices and TDGA between Groups

Correlations between the detected indices in different groups were examined by using heat maps. In the exposed group, IL-8 exhibited strong positive correlations with CXCR2, 8-OHdG, GPX, CAT, MDA, and ROS (*r* = 0.7∼1.0, *P* < 0.001), and a moderate positive correlation with SOD (*r* = 0.6, *P* < 0.05). CXCR2 showed strong positive correlations with IL-8 and 8-OHdG (*r* = 0.7∼1.0, *P* < 0.001) and moderate positive correlations with SOD and ROS (*r* = 0.5, *P* < 0.05). It also demonstrated slight positive correlations with GPX, CAT, and MDA (*r* = 0.2∼0.4), as depicted in Figures 9(d) and 9(e). On the other hand, neither IL-8 nor CXCR2 exhibited significant correlations with any of the indices in the control group in Figures 9(f) and 9(g) (*r* = −0.5∼0.4, *P* > 0.05). Importantly, IL-8 and CXCR2 showed strong positive correlations with each other, indicating their close association with oxidative stress injury under VCM exposure. It was worth noting that no relationship between IL-8, CXCR2, and TDGA was observed.

### 3.11. Correlation of TDGA Content with IL-8, CXCR2, and Oxidative Stress Indices among Posts

**Figure 10:**
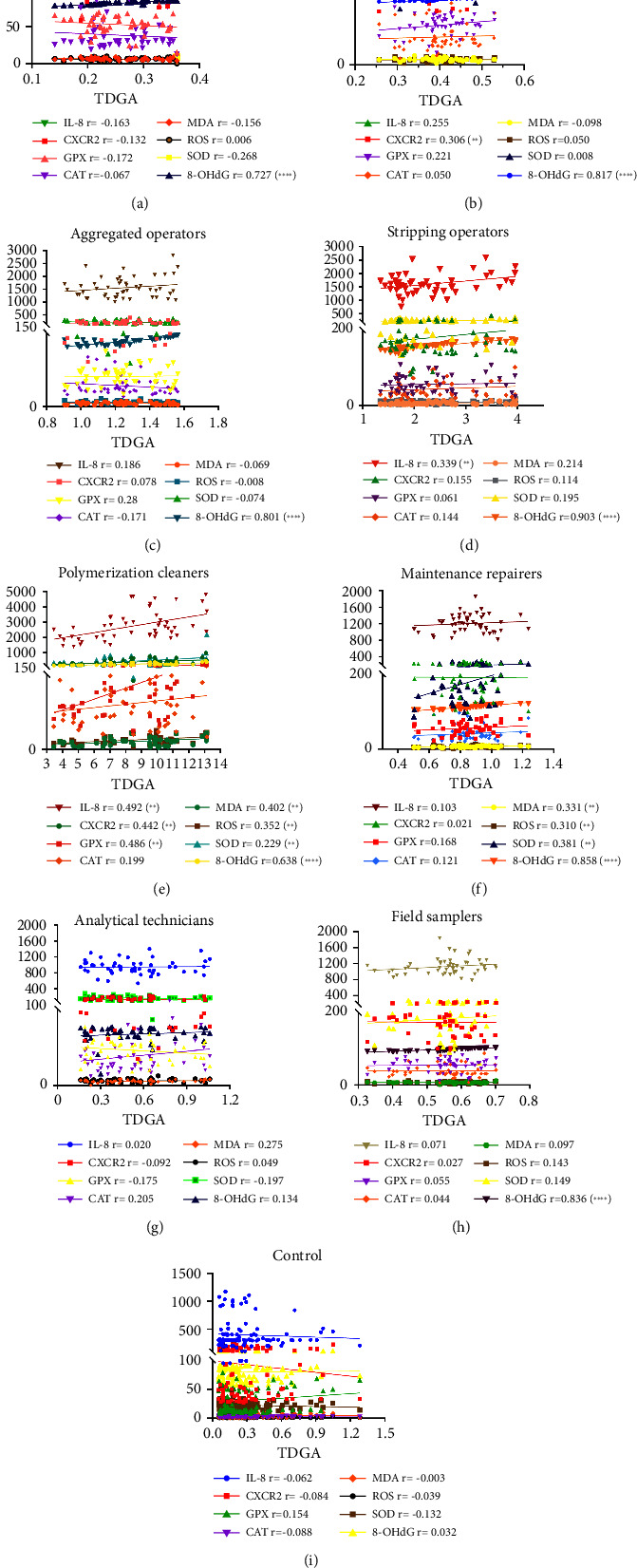
Scatter charts (a–h) presented the correlation intensities of TDGA content with IL-8, CXCR2, 8-OHdG, and other oxidative injury indices among posts in exposed group. *r* represented the correlation intensity coefficient (*r* < 0.4 for mild intensity, 0.4 < *r* < 0.7 for moderate intensity, *r* > 0.7 for strong intensity), ^*∗∗*^*P* < 0.05 and ^*∗∗∗∗*^*P* < 0.001. Chart (a) indicated correlations among indices in synthesis operators; chart (b) indicated correlations among indices in refrigerating operators; chart (c) indicated correlations among indices in aggregated operators; chart (d) indicated correlations among indices in stripping operators; chart (e) indicated correlations among indices in polymerization cleaners; chart (f) indicated correlations among indices in maintenance repairers; chart (g) indicated correlations among indices in analytical technicians; chart (h) indicated correlations among indices in field samplers; chart (i) indicated correlations among indices in operators of the control group.

This section primarily focused on examining whether correlations existed between TDGA content and various indices among different job positions, whether these correlations differed in magnitude. In Figures 10(a), 10(b), 10(c), 10(d), 10(e), 10(f), 10(g), and 10(h), strong positive correlations were observed between TDGA content and 8-OHdG (*r* = 0.638∼0.903, *P* < 0.001) among majority of posts, except for analytical technicians (*r* = 0.134, *P* > 0.05). Refrigerating operators showed weak to moderate positive correlations between TDGA content and IL-8, CXCR2, and GPX levels (*r* = 0.221∼0.306, *P* < 0.05), while polymerization cleaners exhibited moderate positive correlations (*r* = 0.442∼0.492, *P* < 0.05). Conversely, no such correlations were found in the control group, as depicted in Figure 10(i). Therefore, it could be concluded that TDGA content might serve as an effective biomarker for VCM internal exposure status recently and was closely correlated with the extent of oxidative stress injury indicated by 8-OHdG. However, TDGA content did not correlate with IL-8, CXCR2, or other indices related to oxidative stress injury, even among individuals in high-exposure job positions.

### 3.12. Multiple Linear Regression of 8-OHdG and the AST/ALT Ratio between Groups in 2020

Results indicated potential contributions of detected indices towards 8-OHdG and AST/ALT ratio between the two groups. In the exposed group, GPX (*β* = 0.876, 95% CI = 1.224∼1.245), TDGA (*β* = 0.414, 95% CI = 0.566∼1.168), IL-8 (*β* = 0.314, 95% CI = 0.042∼0.144), CXCR2 (*β* = 0.242, 95% CI = 0.128∼0.150), and ROS (*β* = 0.210, 95% CI = 4.020∼4.375) were found to contribute to 8-OHdG content with significant differences (^*∗∗∗∗*^*P* < 0.001 or ^*∗∗*^*P* < 0.05) in positive sequence, while CAT (*β* = −0.418, 95% CI = −1.891∼-1.840), MDA (*β* = −0.404, 95% CI = −1.708∼-1.109), and SOD (*β* = −0.134, 95% CI = −0.032∼-0.044) contributed to 8-OHdG content in an opposite manner. Afterwards, it was amazing to discover that 8-OHdG (*β* = 0.521, 95% CI = 0.080∼0.454), CXCR2 (*β* = 0.452, 95% CI = 0.039∼0.319), MDA (*β* = 0.312, 95% CI = 0.012∼0.025), CAT (*β* = 0.297, 95% CI = 0.012∼0.025), IL-8 (*β* = 0.175, 95% CI = 0.026∼0.259), GPX (*β* = 0.161, 95% CI = 0.044∼0.188), and ROS (*β* = 0.047, 95% CI = 0.030∼0.077) all contributed to AST/ALT ratio with marked differences (^*∗∗∗∗*^*P* < 0.001), respectively, as Table 3 revealed.

In the control group, it was noted that MDA (*β* = −0.463, 95% CI = −7.800∼-1.011) and ROS (*β* = 0.330, 95% CI = 0.078∼6.301) involved in correlation with 8-OHdG content (^*∗∗*^*P* < 0.05), and the contribution made from CXCR2 (*β* = 0.011, 95% CI = 0.067∼0.438) was negligible, as Table 3 revealed. At this point, it could be inferred that IL-8 and CXCR2 in the exposed group obviously reflected the extent of oxidative stress injury and were subsequently related to alterations of AST/ALT ratio in a linear manner under VCM exposure. Thus, the oxidative stress injury might be a key mechanism that intervened in the normal ranges of AST/ALT ratio, where IL-8 and CXCR2 might play potential roles.

### 3.13. Interactive Analysis of Liver *B* Ultrasonography Results between Groups in 2020

**Figure 11:**
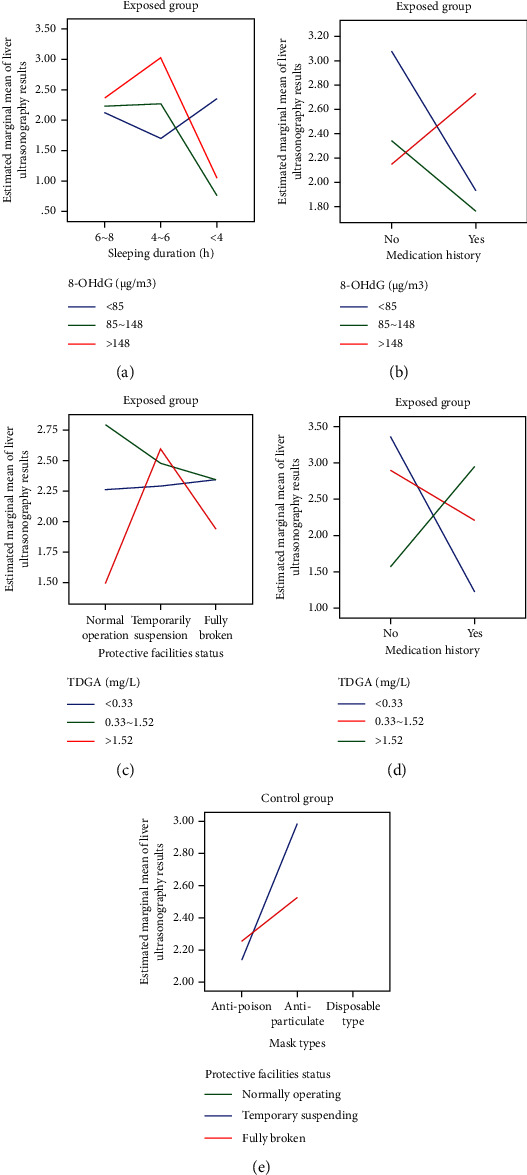
Charts (a–d) presented a series of interactions among 8-OHdG content and sleeping duration, 8-OHdG content and medication history, TDGA content and protective facilities status, and TDGA content and medication history towards liver *B* ultrasonography results in exposed group with significant differences (*P* < 0.05). Chart (e) displayed an interaction between protective facilities status and mask types towards liver *B* ultrasonography results in control group (*P* < 0.05). In that, assignments of liver *B* ultrasonography results were referred to 1 = normal, 2 = abnormal symptoms; of 8-OHdG content referred to 1 ≤ 85, 2 = 85∼148, 3 ≥ 148 *µ*g/m^3^, of TDGA content referred to 1 ≤ 0.33, 2 = 0.33∼1.52, 3 ≥ 1.52 mg/L; of sleeping duration referred to 1 = 6∼8 h, 2 = 4∼6 h, 3 ≤ 4 h; of medication history referred to 1 = no, 2 = yes; of protective facilities status referred to 1 = normal operating, 2 = temporary suspension, 3 = fully broken; of mask types referred to 1 = antipoison, 2 = antiparticulate, 3 = disposable type.

Based on the information presented in Tables 1 and 2, it appeared that variables such as alcohol consumption, working years, mask replacement frequency, mask types, sleeping duration, gender as well as medication history all contributed to liver abnormalities for varying extents. It was worth to explore whether there were any interactive links among these variables. To facilitate binary category analysis, the liver *B* ultrasonography results, originally classified as normal, fatty liver, liver calcification, and liver cysts, were regrouped into “normal” and “abnormal” types. In the exposed group, it was found that content of 8-OHdG (<85, 85∼148, >148 *µ*g/m^3^) interacted with medication history (*F* = 3.376, *P* < 0.05) and sleeping duration (*F* = 5.168, *P* < 0.05), respectively. Specifically, for workers with a definite medication history, content of 8-OHdG exceeding 148 *µ*g/m^3^ was 0.97 times higher than that of <85 *µ*g/m^3^ (95% CI = 0.196∼1.823, *P* < 0.05). Furthermore, for 8-OHdG content exceeding 148 *µ*g/m^3^, workers with an average sleeping duration of 4–6 hours had a 0.83-fold higher risk as compared to those with a sleeping duration of 6–8 hours (95% CI = 0.252∼1.406, *P* < 0.05). Content of TDGA (<0.33, 0.33∼1.52, >1.52 mg/L) also interacted with the status of protective facilities (*F* = 3.035, *P* < 0.05) and medication history (*F* = 5.448, *P* < 0.05). Particularly, for workers who considered protective facilities to be in normal operating status, risks associated with TDGA content of 0.33∼1.52 mg/L were 1.3 times higher than those associated with >1.52 mg/L (95% CI = 0.402∼2.199, *P* < 0.05). When TDGA content exceeded 1.52 mg/L, risks for workers identifying temporary suspension were 1.1 times higher than those considering it as normal operating (95% CI = 0.393∼1.801, *P* < 0.05). Additionally, for workers with a definite medication history, risks associated with TDGA content exceeding 1.52 mg/L were 1.7 times higher than those associated with <0.33 mg/L (95% CI = 0.528∼2.917, *P* < 0.05) as presented in Figures 11(a), 11(b), 11(c), and 11(d). In the control group, the only interactive link observed was between mask type and status of protective facilities (*F* = 2.645, *P* < 0.05). For workers who usually wore antiparticulate masks, risks with ones who regarding temporarily suspended facilities were 0.68 times higher than for those confirmed as fully functional (95% CI = 0.241∼1.636, *P* < 0.05), as Figure 11(e) presented. It could be inferred that high levels of 8-OHdG (>148 *µ*g/m^3^) interacted with a definite medication history and shorter sleeping duration (4–6 hours) to contribute to liver abnormalities as oxidative stress injury. Furthermore, TDGA content (>1.52 mg/L) also interacted with the status of protective facilities on temporary suspension and a definite medication history, particularly when VCM concentration reached relatively high levels. Inadequate protective facilities and inappropriate mask types might also increase the risks of liver abnormalities through reciprocal interactions.

### 3.14. Interactive Analysis of the AST/ALT Ratio between Groups in 2020

Based on findings presented in Figures 5 and 6, it was evident to note that the AST/ALT ratio played a significant role in determining the binary categorization of liver as “normal” or “abnormal” in consecutive years from 2012 to 2018, as well as in specific abnormality categories such as “normal,” “fatty liver,” “liver calcification,” and “liver cysts” in 2020. It was of interest to explore any potential interactions between IL-8, CXCR2, 8-OHdG, TDGA, and variables from questionnaire and how these interactions correlated with the AST/ALT ratio through a general linear model under pairwise comparison analysis.

**Figure 12:**
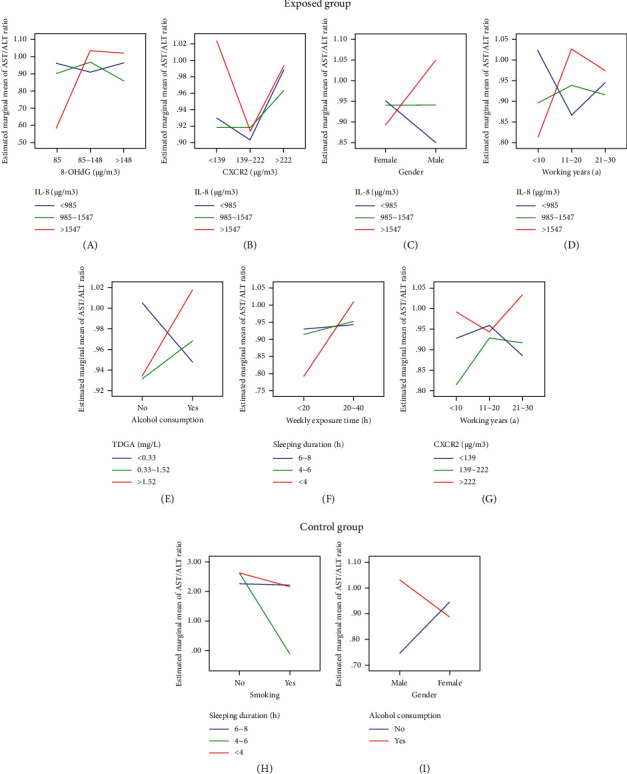
Charts (A–G) demonstrated interactions among content of IL-8, CXCR2, 8-OHdG, TDGA, and questionnaire variables towards AST/ALT ratio in the exposed group. Charts (A–D) presented variable interactions of IL-8 content with 8-OHdG, CXCR2, gender, and working years (a); charts (E and F) indicated interactions of TDGA content with alcohol consumption, and sleeping duration with weekly exposure time (h); charts (G) indicated interactions of CXCR2 content with working years (a). Charts (H and I) displayed interactions between sleeping duration and smoking, alcohol consumption and gender toward AST/ALT ratio in the control group. Particularly, the assignment of 8-OHdG content referred to 1 ≤ 85, 2 = 85∼148, 3 ≥ 148 *µ*g/m^3^, of TDGA content referred to 1 ≤ 0.33, 2 = 0.33∼1.52, 3 ≥ 1.52 mg/L; of IL-8 content referred to 1 ≤ 985, 2 = 985∼1547, 3 ≥ 1547 *µ*g/m^3^; of CXCR2 content referred to 1 ≤ 139, 2 = 139∼222, 3 ≥ 222 *µ*g/m^3^; of alcohol consumption referred to 1 = no, 2 = yes; of gender referred to 1 = male, 2 = female; of sleeping duration referred to 1 = 6∼8 h, 2 = 4∼6 h, 3 ≤ 4 h; of smoking referred to 1 = no, 2 = yes; of working years referred to 1 ≤ 10(a), 2 = 11∼20(a), 3 = 21∼30(a).

In the exposed group, as depicted in Figures 12(A), 12(B), 12(C), and 12(D), it was observed that IL-8 (<985, 985∼1547, >1547 *µ*g/m^3^) interacted with 8-OHdG (<85, 85∼148, >148 *µ*g/m^3^), CXCR2 (<139, 139∼222, >222 *µ*g/m^3^), gender, and working years (<10a, 11∼20a, 21∼30a) in relation to the AST/ALT ratio with significant differences (*P* < 0.05). Specifically, for workers with IL-8 content exceeding 1547 *µ*g/m^3^, 8-OHdG content ranging from 85 to 148 *µ*g/m^3^ was 0.62 times higher than that below of 85 *µ*g/m^3^ (95% CI = 0.198∼1.519, *P* < 0.05). Similarly, CXCR2 content lower than 139 *µ*g/m^3^ was 0.12 times higher than that ranging from 139 to 222 *µ*g/m^3^ (95% CI = 0.602∼1.019, *P* < 0.05). Male workers exhibited a 0.18 times higher ratio as compared to female workers (95% CI = 0.439∼1.288, *P* < 0.05), and those with working years ranging from 11 to 20 (a) had a 0.22 times higher ratio than those with less than 10 (a) (95% CI = 0.327∼1.287, *P* < 0.05). Notably, CXCR2 also showed an interaction with working years, particularly when the content exceeded 222 *µ*g/m^3^. Working years ranging from 21 to 30 (a) were 0.11 times higher than those ranging from 11 to 20 (a) (95% CI = 0.222∼1.424, *P* < 0.05). Additionally, there were other interactions affecting the AST/ALT ratio, including TDGA content (<0.33, 0.33∼1.52, >1.52 mg/L), alcohol consumption, sleeping duration (6∼8, 4∼6, <4 h), and working exposure time (<20, 20∼40 h). For instance, when workers' TDGA content exceeded 1.52 mg/L, those who consumed alcohol had a 0.10 times higher ratio than those who never drank alcohol before (95% CI = 0.041∼1.246, *P* < 0.05). Similarly, for workers with a sleeping duration of less than 4 hours per day, those exposed to VCM for 20–40 hours weekly had a 0.22 times higher ratio than those exposed for less than 20 hours (95% CI = 0.245∼1.481, *P* < 0.05), as depicted in Figures 12(E), 12(F), and 12(G).

Surprisingly, in the control group, sleeping duration (6∼8, 4∼6, <4 h) interacted with smoking habits, while alcohol consumption interacted with gender in relation to the AST/ALT ratio with notable differences (*P* < 0.05). Specifically, for workers who usually slept less than 4 hours per day, nonsmokers had a 2.90 times higher ratio compared to smokers (95% CI = 0.0232∼4.328, *P* < 0.05). Furthermore, for workers who consumed alcohol, male workers had a 0.23 times higher ratio than female workers (95% CI = 0.027∼1.128, *P* < 0.05). This suggested that unhealthy lifestyle habits such as alcohol consumption and insufficient sleeping duration may be detrimental to maintaining a normal range of AST/ALT ratios, as shown in Figure 12(H) and I. It could be inferred that both IL-8 (>1547 *µ*g/m^3^) and CXCR2 (<139 *µ*g/m^3^) contributed to the AST/ALT ratio through reciprocal interactions under oxidative stress injury. Furthermore, factors such as CXCR2 (>222 *µ*g/m^3^), longer working years of 21 to 30 (a) or 11 to 20 (a), TDGA content (>1.52 mg/L), definite alcohol consumption, smoking habits, shorter sleeping duration of <4 hours per day, and being male also played potential roles more or less.

## 4. Discussion

Numerous studies have indicated that high levels of occupational exposure to VCM will lead to liver damage, which is often characterized by hepatocellular injury and functional disturbances. By the time liver abnormalities are detected through imaging or abnormal indicators, it may already be too late for urgent clinical treatment [24]. This study observed various changes in liver function indicators (ALT, AST, and AST/ALT ratio), results from liver *B* ultrasonography tests (including fatty liver, liver calcification, and liver cysts), detected indices for inflammatory factors such as IL-8 and CXCR2, as well as indices concerning oxidative stress injury. These findings suggested that these factors could potentially be utilized for screening liver abnormalities prior to liver impairment.

The results of the retrospective survey revealed that liver abnormalities caused by VCM exposure were consistently present across different job posts. Levels of ALT, AST, AST/ALT ratio, and *γ*-GGT were higher in workers with “abnormal” symptoms compared to those with “normal” symptoms, even improvement measures on practical reductions in VCM concentration had taken place. Furthermore, the composition ratios (*r*%) of “abnormal symptoms” indicated a continuous tendency towards liver impairment over years. These findings suggested that the reduction in VCM concentration did not prevent liver abnormalities from progression and development. In fact, improvement measures were already being implemented in VCM manufacturing facilities to reduce VCM concentration from a preliminary survey. Previous results from our study [25] showed a significant decrease in abnormal hepatic symptoms in the majority of posts at the PVC factory (Y) that employed the acetylene hydrochlorination technique, as compared to another factory that using the ethylene oxychlorination technique in Tianjin City. Improvements, such as enhanced ventilation and air-tightness, also showed notable effects on health indicators related to fatty liver and other hepatic symptoms in both factories (Y and Z), including ALT, AST, and *γ*-GGT levels. These findings indicated that changes in these indices partially reflected the status of liver impairment and the effectiveness of the improvement measures. In particular, the volatile gas released from VCM, especially originated from open observation ports, was controlled through emergency ventilation facilities with increased exhaust air and ventilation air changing rates. Binary logistic regression analysis from 2012 to 2018 and multiple logistic regression analysis in 2020 all demonstrated that indicators of ALT, AST, and AST/ALT ratio could serve as prominent indicators for screening liver abnormalities in the initial phase. Various domestic and international studies have shown that the aminotransferase levels or AST/ALT ratio could be potential biomarkers for liver functional impairment, including the incidence of liver cancer and abnormalities in metabolic functions, such as hyperlipidemia and hyperglycemia [21]. In fact, the concept of the aspartate transaminase/alanine transaminase (AST/ALT) ratio is initially proposed for the study of hepatitis etiology and is commonly used to differentiate different causes of liver disease, such as fatty liver. It is considered an effective biomarker for liver disease screening, especially when the AST/ALT ratio exceeds the limit of 1.15, which is a clinical and preventive standard for cardiovascular diseases, various cancers even type 2 diabetes mellitus [26]. A high AST/ALT ratio (>1.15) has also been associated with predicting poor prognosis in nonmetastatic renal cell carcinoma. For instance, a study with 9,946 participants conducted by the BaiYun community Health Service Center in Taizhou, Jiangsu Province, China (2015–2019), revealed significant correlations between elevated AST/ALT ratio and increased mortality risks (OR = 1.495, *P* < 0.05, 95% CI = 1.003-2.228) after adjusting for demographic and behavioral variables [27]. The AST/ALT ratio has also been identified as an independent risk factor for forecasting population health risks, positive associations have been observed between blood pressure and AST/ALT ratio with diastolic blood pressure. Furthermore, the AST/ALT ratio has been advocated for cancer development. For example, Li et al. found that a higher AST/ALT ratio was an independent predictor of 1-year mortality in polymyositis-/dermatomyositis-associated interstitial lung disease, and it was associated with poor outcomes in renal cell carcinoma, head and neck carcinoma, oral and oropharyngeal carcinoma, and other malignant cancers [28]. Based on these findings, the AST/ALT ratio could be considered as an effective biomarker for identifying liver abnormalities caused by VCM during the initial phase, supported by abundant evidence.

Furthermore, the 2020 VCM concentration detection revealed a strong correlation between higher concentrations in the exposed group and their relevant TDGA content. Specifically, posts occupied by aggregated operators, stripping operators, polymerization cleaners, maintenance repairers, and field samplers had concentrations within the range of 50% to 1 fold OEL or exceeded 1 fold OEL. This indicated that workers were potentially at risk of health, and requirements to reduce ambient VCM exposure levels immediately were urgent. During field investigations, several vulnerabilities in VCM production facilities were identified, particularly in the maintenance of protective measures, operation of airtight techniques, and implementation of warning notices. These three factors were the main contributors to occupational health risks, particularly, it usually involved in insufficient emergency ventilation systems, malfunctioning ventilation equipment, inadequate indoor air distribution, improper setup of exhaust hoods, uncovered observation ports, unsealed valves or cover plates, and absence of warning lines and enclosures. All of these systematically affected the diffusion and spatial distribution of VCM concentration, ultimately compromising the health status of workers. Subsequently, odds ratio (OR) values gradually increased when grouping specific categories related to liver calcification, liver cysts, and fatty liver. This finding suggested a hypothesis that the increased VCM concentration among workers may be a primary cause of the rising incidence of liver abnormalities.

Detection on IL-8, CXCR2, and other oxidative stress injury indices revealed that increased exposure to VCM could elevate the levels of IL-8 and CXCR2 and intensify their correlation among workers through oxidative stress injury mechanisms. Studies conducted by Chen [29] and Nguyen NT [30] have shown that IL-8 typically binds tightly to CXCR2 and this IL8-CXCR2 axis plays a pivotal role in liver inflammation induction. It recruits neutrophils to infection or injury sites, where they primarily clear pathogens, cellular debris, alarmins, and metabolic waste. However, this process can also induce inflammation injury and lead to hepatocyte death. Once neutrophils reach the tissues, CXCR2 activation triggers the release of granule enzymes and reactive oxygen species (ROS), which aid in pathogen elimination and up-regulate IL-8 expression. The infiltration and accumulation of immune cells have been found to correlate significantly with chronic inflammation in the liver, based on these, IL-8 and its ligand CXCR2 were selected as the breakthrough point.

Additionally, multiple logistic linear analyses suggested that IL-8, CXCR2, and 8-OHdG could influence the AST/ALT ratio under VCM exposure. Interaction analysis also indicated that high levels of IL-8 (>1547 *µ*g/m^3^) or low levels of CXCR2 (<139 *µ*g/m^3^) were associated with changes in the AST/ALT ratio under oxidative stress injury. Moreover, other interactions were observed between CXCR2 (>222 *µ*g/m^3^) and longer working years of 21 to 30 (a) or 11 to 20 (a), as well as high TDGA content (>1.52 mg/L) and alcohol consumption. It could be assumed that long-term sleep disorders, irregular insomnia, and factors such as spiritual pressure, heavy workload, disturbed biological clocks due to long working years, and even occupational psychological stress induced by environmental noise or high temperatures all probably contributed to liver impairment and might exacerbated occupational health risks.

Interaction analysis results for liver ultrasonography tests, categorized as abnormal and normal, showed that high levels of 8-OHdG (>148 *µ*g/m^3^) interacted with a definite medication history and shorter sleeping duration (4∼6 hours) in contributing to liver abnormalities. Additionally, TDGA content (>1.52 mg/L) was noted to interact with the presence of protective facilities during temporary suspension and a definite medication history, particularly when VCM concentration reached relatively high levels. Inadequate protective facilities and the use of inappropriate mask types also increased the risk of liver abnormalities through reciprocal interactions. It could be noted that factors such as insufficient protective facilities, lack of proper warning closures, longer exposure time at risky points, frequent shift work, fatigue, restricted operating and patrolling areas, inadequate surveillance of correct mask types as well as failure to replace masks effectively contributed to increased exposure levels and diminished awareness of self-safety protection gradually.

Specifically, liver abnormalities, such as liver calcification, multiple cysts, and moderate to severe fatty liver, were considered initial symptoms of nonalcoholic fatty liver disease (NAFLD), as NAFLD encompassed simple steatosis and nonalcoholic steatohepatitis (NASH), which could progress to fibrosis, cirrhosis, and hepatocellular carcinoma (HCC) [31]. Excessive accumulation of fat in liver of patients without significant alcohol consumption was a major cause of liver dysfunction and chronic liver disease worldwide. In an effort to raise awareness of the disease, the definition and nomenclature of NAFLD had been changed to metabolic dysfunction-associated fatty liver disease (MAFLD). An international panel of experts from 22 countries had proposed diagnostic criteria for MAFLD, taking into account disease heterogeneity and the role of underlying metabolic factors in disease development. MAFLD was diagnosed in patients with evidence of hepatic steatosis (based on imaging, histopathological examination, or blood biomarker testing) and the presence of at least one of the following three metabolic criteria: obesity/overweight, established type 2 diabetes mellitus (T2DM), or metabolic dysregulation [32]. Numerous studies had identified the association of NAFLD with insulin resistance, metabolic syndrome, diabetes mellitus, and obesity, which led to intrahepatic triglyceride accumulation, increased production of reactive oxygen species, oxidative stress, as well as lipid peroxidation [33].

According to the multiple-hit hypothesis, insulin resistance played a critical role in the pathogenesis of NAFL/NASH. It led to an increased hepatic *de novo* lipogenesis and dysfunction of adipose tissue, resulting in elevated levels of circulating free fatty acids. These in turn impaired the secretion of adipokines and inflammatory cytokines such as IL-6, IL-8, tumor necrosis factor-*α* (TNF-*α*) and adiponectin [34]^.^ Based on our results, it appeared that oxidative stress injury caused by VCM exposure was an important mechanism for liver abnormalities during the early screening phase. Abnormal changes in AST/ALT ratio in the blood should be identified as a specific indicator for initial screening, especially in conjunction with liver *B* ultrasonography results. IL-8 and CXCR2 had been shown to contribute to the AST/ALT ratio affection, especially when reached to high levels. Other variables such as longer working years (21 to 30 or 11 to 20), alcohol consumption, shorter sleeping duration (4∼6 hours or less than 4 hours per day), definite medication history, inadequate protective facilities, and inappropriate mask types were also influential factors that were to be considered comprehensively.

## 5. Conclusions

This study made several significant findings. Firstly, it revealed that even with a decrease in VCM concentration among posts in PVC manufacturing factories, liver abnormalities, primarily characterized by fatty liver, liver calcification, and liver cysts, could still occur and progressed, due to oxidative stress injury, in which participation of IL-8 and CXCR2 were involved. Secondly, the AST/ALT ratio was regarded as a specific indicator that could help detecting liver abnormalities before impairment, especially when combined with liver *B* ultrasonography results. Third, IL-8 and CXCR2 were noted to correlate strongly with each other and contributed to the alteration of the AST/ALT ratio under VCM exposure. Lastly, factors like definite medication history, inadequate protective facility status (fully broken), alcohol consumption, shorter sleeping duration (4–6 hours and less than 4 hours per day), inappropriate mask types, and longer working years of 21 to 30 (a) or 11 to 20 (a), could contribute to changes in the AST/ALT ratio through complex interactions. Overall, these findings highlighted the importance on mechanisms underlying liver abnormalities and the significance of AST/ALT ratio as an early indicator and the influence of various variables on liver health.

## 6. Limitations

In this study, it is important to acknowledge several limitations that should be considered for future research. Firstly, the sample sizes of the VCM-exposed population were chosen solely from a PVC factory with acetylene hydrochlorination technique, while workers from the ethylene oxychlorination technique were not contained in this study, which would limit the generalization performance to other settings or populations that are affected by VCM. Secondly, results of liver *B* ultrasonography tests conducted from 2012 to 2018 were categorized as either “normal” or “abnormal” without further detailed analysis on distribution discrepancies and trends of abnormal symptoms among specific job posts due to data deficiency. This restricted our ability to perform continuous analysis on these aspects. Thirdly, the distribution and difference analysis of abnormal symptoms among job positions in Group G were compromised due to the unavailability of matched results in 2020. This limitation prevented us from fully examining the correlations and interactions between abnormal symptoms and job positions within this specific group. Fourthly, no such elaborated analysis was taken to conduct the differential results among groups stratified through alcohol consumption, such as weekly frequency of alcohol drinking, aging of alcohol consumption, and these regrets will be accomplished in near further. Lastly, the factual causes towards correlation among IL-8, CXCR2, and the AST/ALT ratio were not deeply explored, which will certainly become our critical point in near future, it is important to note that the correlations and interactions observed between IL-8, CXCR2, and other detected indices and variables from questionnaire were only proven on a statistical level. Further assessment is needed to establish their practical relationships with each other. Addressing these limitations in future studies will provide a more comprehensive understanding of the factors influencing the outcomes observed in this study.

## Figures and Tables

**Table 1:** Questionnaire data analysis between two groups under symptom categories of liver *B* ultrasonography test results in 2020.

Variables	Exposed group (*n* = 227)								*t/X* ^2^,*P* value^1^	Control group (*n* = 110)								*t/X* ^2^,*P* value^2^	*t/X* ^2^, *P* value^3^
	Normal (*n* = 104)		Fatty liver (*n* = 49)		Liver calcification (*n* = 38)		Liver cysts (*n* = 36)			Normal (*n* = 49)		Fatty liver (*n* = 33)		Liver calcification (*n* = 16)		Liver cysts (*n* = 12)			
	*n*	*r*(%)	*n*	*r*(%)	*n*	*r*(%)	*n*	*r*(%)		*n*	*r(%)*	*n*	*r(%)*	*n*	*r(%)*	*n*	*r(%)*		
Age (a), Mean ± SD																			
Male (*n* = 209 *vs n* = 70)	43.52 ± 4.24								3.23, >0.05	39.54 ± 3.21								2.92, >0.05	4.52,<0.001^*∗∗∗∗*^
Female (*n* = 18 *vs n* = 40)	42.12 ± 2.07									40.06 ± 4.08									2.84, >0.05
Working years (a), Mean ± SD																			
Male (*n* = 209 *vs n* = 70)	21.79 ± 5.16								4.37, >0.05	19.69 ± 4.26								4.81, >0.05	6.98,<0.001^*∗∗∗∗*^
Female (*n* = 18 *vs n* = 40)	20.57 ± 3.22									16.68 ± 5.09									8.69,<0.001^*∗∗∗∗*^
Gender																			
Male	97	93.3	43	87.8	36	94.7	32	88.9	2.16, >0.05	28	57.1	26	78.8	8	50.0	8	66.7	5.50, >0.05	7.95, <0.05^*∗∗*^
Female	7	6.70	6	12.2	2	5.3	4	11.1		21	42.9	7	21.2	8	50.0	4	33.3		3.67, >0.05
Age range (a)																			
∼30	22	21.2	13	26.5	9	23.7	12	33.3	7.47, >0.05	21	42.9	7	21.2	2	12.5	1	8.3	12.13 > 0.05	9.16, <0.05^*∗∗*^
∼45	49	47.1	20	40.8	14	36.8	8	22.2		14	28.6	12	36.4	4	25.0	5	41.7		3.49, >0.05
∼60	33	31.7	16	32.7	15	39.5	16	44.4		14	28.6	14	42.4	10	62.5	6	50.0		3.18, >0.05
Medication history																			
Yes	39	37.5	20	40.8	12	31.6	8	22.2	3.80, >0.05	13	26.5	20	60.6	13	81.3	7	58.3	18.74, <0.001^*∗∗∗∗*^	7.85, <0.05^*∗∗*^
No	65	62.5	29	59.2	26	68.4	28	77.8		36	73.5	13	39.4	3	18.8	5	41.7		2.99, >0.05
Smoking habits																			
Yes	45	43.3	14	28.6	22	57.9	8	22.2	12.97, <0.05^*∗∗*^	19	38.8	20	60.6	4	25.0	5	41.6	6.62, >0.05	13.73,<0.05^*∗∗*^
No	59	56.7	35	71.4	16	42.1	28	77.8		30	61.2	13	39.4	12	75.0	7	58.3		4.47, >0.05
Alcohol consumption																			
Yes	39	37.5	12	24.5	9	23.7	11	30.6	3.95, >0.05	22	44.9	9	27.3	8	50.0	4	33.3	3.60, >0.05	1.72, >0.05
No	65	62.5	37	75.5	29	76.3	25	69.4		27	55.1	24	72.7	8	50.0	8	66.7		4.30, >0.05
Working years (a)																			
∼10	26	25.0	9	18.4	5	13.2	4	11.1	15.85, <0.05^*∗∗*^	16	32.7	9	27.3	4	25.0	2	16.7	11.19, >0.05	0.93, >0.05
∼20	50	48.1	13	26.5	20	52.6	18	50.0		23	46.9	12	36.4	3	18.8	3	25.0		9.68,<0.05^*∗∗*^
∼30	28	26.9	27	55.1	13	34.2	14	38.9		10	20.4	12	36.4	9	56.3	7	58.3		1.41, >0.05
Sleeping duration (h)																			
∼4	24	23.1	8	16.3	11	28.9	10	27.8	8.57, >0.05	5	10.2	2	6.1	6	37.5	4	33.3	20.34, <0.05^*∗∗*^	2.14, >0.05
∼6	59	56.7	23	46.9	14	36.8	17	47.2		31	63.3	13	39.4	8	50.0	5	41.7		1.37, >0.05
∼8	21	20.2	18	36.7	13	34.2	9	25.0		13	26.5	18	54.5	2	12.5	3	25.0		6.97, >0.05
Weekly exposure time (h)																			
∼20	46	44.2	23	46.9	12	31.6	24	66.7	9.61, <0.05^*∗∗*^	8	16.3	12	36.4	4	25.0	4	33.3	4.62, >0.05	5.87, >0.05
∼40	58	55.8	26	53.1	26	68.4	12	33.3		41	83.7	21	63.6	12	75.0	8	66.7		1.63, >0.05
Mask types																			
Antipoison	72	69.2	16	32.7	11	28.9	8	22.2	44.78, <0.001^*∗∗∗∗*^	8	16.3	7	21.2	2	12.5	2	16.7	12.35, >0.05	6.04, >0.05
Antiparticulate	28	26.9	30	61.2	22	57.9	20	55.6		2	4.1	6	18.2	6	37.5	2	16.7		3.31, >0.05
Disposable	4	3.85	3	6.12	5	13.2	8	22.2		39	75.6	20	60.6	8	50.0	8	66.7		14.90,<0.05^*∗∗*^
Frequency of wearing																			
Always	69	66.3	21	42.9	16	42.1	13	36.1	15.34, <0.05^*∗∗*^	29	59.2	13	39.4	10	62.5	8	66.7	6.32, >0.05	1.31, >0.05
Certain times	35	33.7	28	57.1	22	57.9	23	63.9		21	42.9	20	60.6	6	37.5	2	16.7		4.78, >0.05
Mask replacement frequency																			
Once per day	27	26.0	18	36.7	13	34.2	19	52.8	8.82, <0.05^*∗∗*^	16	32.7	11	33.3	8	50.0	7	58.3	3.98, >0.05	1.03, >0.05
Infrequently	77	74.0	31	63.3	25	65.8	17	47.2		33	67.3	22	66.7	8	50.0	5	41.7		4.18, >0.05
Protective facilities status																			
Normally operating	72	69.2	14	28.6	7	18.4	5	13.9	65.07 < 0.001^*∗∗∗∗*^	46	93.9	29	87.9	14	87.5	11	91.7	2.09, >0.05	15.53,<0.05^*∗∗*^
Temporarily suspending	25	24.0	29	59.2	21	55.3	29	80.6		2	4.1	4	12.1	2	12.5	1	8.3		1.68, >0.05
Fully broken	7	6.70	6	12.2	10	26.3	2	8.30		1	2.0	0	0	0	0	0	0		—

*Note*. The population size (*n*) and the composition ratio (*r*%) were reported in the following paragraph. Workers from both groups were classified into different categories based on their liver *B* ultrasonography results in 2020: “normal” (*n* = 104; *n* = 49), “fatty liver” (*n* = 49; *n* = 33), “liver calcification” (*n* = 38; *n* = 16), and “liver cysts” (*n* = 36; *n* = 12). Statistical analysis was conducted using the chi-square test to examine the association between symptom categories and all variables in the exposed group (denoted by^1^). Similarly, for the control group, statistical analysis was performed to assess the relationship between abnormality categories and all variables (denoted by^2^). Furthermore, the chi-square test was used to analyze the differences in abnormality categories between the two groups across each stratified factor and all variables (denoted by^3^). Significance levels were indicated by^*∗∗*^*P* < 0.05 and ^*∗∗∗∗*^*P* < 0.001.

**Table 2:** Multiple logistic regression analysis for liver *B* ultrasonography results between groups in 2020.

Liver symptom categories	Variables	*B* value	Wald	*P* value	Exp(*B*)	95% CI
Fatty liver^1^	*Alcohol consumption*					
	No	1				
	Yes	1.044	2.996	0.033^*∗∗*^	1.288	1.071∼2.179
	*Working years (a)*					
	∼10	1				
	∼20	1.374	3.298	0.026^*∗∗*^	1.488	1.161∼2.310
	∼30	1.477	3.809	0.014^*∗∗*^	1.626	1.252∼2.861

Liver calcification^1^	*Alcohol consumption*					
	No	1				
	Yes	0.831	3.367	0.024^*∗∗*^	1.592	1.277∼3.063
	*Working years (a)*					
	∼10	1				
	∼20	0.209	2.684	0.037^*∗∗*^	1.116	1.020∼1.420
	∼30	0.341	2.011	0.035^*∗∗*^	1.406	1.043∼1.895
	*Mask replacement frequency*					
	Once per day	1				
	Infrequently	0.119	3.641	0.018^*∗∗*^	1.004	0.932∼1.321
	*Mask types*					
	Antipoison	1				
	Antiparticulate	1.015	2.035	0.154	1.133	0.798∼2.124
	Disposable type	1.644	3.216	0.028^*∗∗*^	1.026	0.901∼1.906
	*Sleeping duration (h)*					
	∼8	1				
	∼6	0.388	3.527	0.016^*∗∗*^	2.059	1.129∼4.615
	∼4	0.411	3.292	0.023^*∗∗*^	1.249	1.154∼3.801

Liver cysts^1^	*Alcohol consumption*					
	No	1				
	Yes	0.744	2.765	0.040^*∗∗*^	1.801	1.030∼3.483

Fatty liver^2^	*Gender*					
	Female	1				
	Male	0.518	2.447	0.037^*∗∗*^	1.047	0.971∼1.129
	*Alcohol consumption*					
	No	1				
	Yes	0.934	2.544	0.036^*∗∗*^	2.421	1.141∼4.003

Liver calcification^2^	*Medication history*					
	No	1				
	Yes	1.113	2.806	0.028^*∗∗*^	1.329	1.067∼1.620
	*Alcohol consumption*					
	No	1				
	Yes	0.884	3.172	0.014^*∗∗*^	1.761	1.161∼3.323

*Note*. ^1^presented the exposed group, ^2^presented the control group. Assignment toward independent variables with significant contributions were elaborated as follows: alcohol consumption (1 = no, 2 = yes); working years (1 = ∼10a, 2 = ∼20a, 3 = ∼30a); mask replacement frequency (1 = once per day, 2 = infrequently); mask types (1 = antipoison, 2 = antiparticulate, 3 = disposable type); sleeping duration (1 = ∼8 h, 2 = ∼6 h, 3 = ∼4 h); gender (1 = female, 2 = male); medication history (1 = no, 2 = yes), ^*∗∗*^presented *P* < 0.05.

**Table 3:** Multiple linear regression analysis of 8-OHdG content and AST/ALT ratio in 2020.

Variables	Exposed group (*n* = 227)				Control group (*n* = 110)			
	*β*	*t*	*P* value	95% CI	*β*	*t*	*P* value	95% CI
IL-8^1^	0.314	78.93	<0.001^*∗∗∗∗*^	0.042∼0.144	0.127	0.99	0.327	0.019∼0.027
CXCR2^1^	0.242	24.46	<0.001^*∗∗∗∗*^	0.128∼0.150	0.011	2.75	0.009^*∗∗*^	0.067∼0.438
GPX^1^	0.876	223.40	<0.001^*∗∗∗∗*^	1.224∼1.245	0.103	1.22	0.228	0.347∼1.006
CAT^1^	−0.418	−142.85	<0.001^*∗∗∗∗*^	−1.891∼−1.840	−0.119	−1.31	0.199	−0.516∼0.111
MDA^1^	−0.404	−81.12	0.003^*∗∗*^	−1.708∼−1.109	−0.463	−2.62	0.012^*∗∗*^	−7.800∼−1.011
ROS^1^	0.210	46.33	0.009^*∗∗*^	4.020∼4.375	0.330	2.07	0.045^*∗∗*^	0.078∼6.301
SOD^1^	−0.134	−11.47	0.014^*∗∗*^	−0.032∼−0.044	−0.171	−1.84	0.074	−0.091∼0.004
TDGA^1^	0.414	33.62	<0.001^*∗∗∗∗*^	0.566∼1.168	0.005	1.45	0.150	0.002∼0.013

8-OHdG^2^	0.521	34.42	<0.001^*∗∗∗∗*^	0.080∼0.454	0.176	0.93	0.354	0.002∼0.055
IL-8^2^	0.175	17.17	<0.001^*∗∗∗∗*^	0.026∼0.259	0.001	0.01	0.996	0.011∼0.046
CXCR2^2^	0.452	29.23	<0.001^*∗∗∗∗*^	0.039∼0.319	0.102	0.63	0.529	0.004∼0.029
GPX^2^	0.161	8.28	<0.001^*∗∗∗∗*^	0.044∼ 0.188	0.043	0.35	0.725	0.002∼0.048
CAT^2^	0.297	24.18	<0.001^*∗∗∗∗*^	0.178∼0.639	0.079	0.72	0.473	0.016∼0.034
MDA^2^	0.312	15.95	<0.001^*∗∗∗∗*^	0.012∼0.025	0.066	0.25	0.803	0.017∼0.039
ROS^2^	0.074	4.58	<0.001 ^*∗∗∗∗*^	0.030∼0.077	0.183	1.34	0.185	−0.101∼0.021
SOD^2^	−0.017	−1.60	0.110	−0.116∼0.044	−0.101	−0.92	0.363	0.007∼0.033
TDGA^2^	0.002	0.54	0.590	0.002∼0.024	0.105	0.96	0.340	0.007∼0.028

*Note*. *β* presented the standardized coefficient, it indicated the contributions to the dependent variable after standardization among variables at different quantities. In that, ^1^presented 8-OHdG content as the dependent variable in this linear regression model, while ^2^presented as AST/ALT ratio, as^*∗∗∗∗*^indicated *P* < 0.001, ^*∗∗*^indicated *P* < 0.05.

## Data Availability

The original contributions presented in the study are included in the article/supplementary material; further inquiries can be directed to the corresponding author. Data concerning physical examination indicators, health archives, and occupational history are only available in the occupational physical examination center and the department of occupational health and radiological health, center for disease control and prevention in Bin Hai New Area of Tian Jin City; detection results and processing charts concerning field investigation as well as layout settings are only available in G and H. However, restrictions apply to the availability of these data and might not be publicly available.
